# New electroactive asymmetrical chalcones and therefrom derived 2-amino- / 2-(1*H*-pyrrol-1-yl)pyrimidines, containing an *N*-[ω-(4-methoxyphenoxy)alkyl]carbazole fragment: synthesis, optical and electrochemical properties

**DOI:** 10.3762/bjoc.13.158

**Published:** 2017-08-10

**Authors:** Daria G Selivanova, Alexei A Gorbunov, Olga A Mayorova, Alexander N Vasyanin, Igor V Lunegov, Elena V Shklyaeva, Georgii G Abashev

**Affiliations:** 1 Laboratory of Active Reagents Synthesis, Institute of Technical Chemistry, Russian Academy of Sciences, Ural Division, Academician Korolev street, 3, 614990, Perm, Russia; 2 Department of Analytical Chemistry, Perm State University, Bukirev street, 15, 614990, Perm, Russia; 3 Department of Physics, Radio Electronics and Information Security, Perm State University, Bukirev Street, 15, 614990, Perm, Russia; 4 Department of Organic Chemistry, Perm State University, Bukirev street, 15, 614990, Perm, Russia; 5 Laboratory of organic semiconductors, Natural Sciences Institute, Perm State University, Genkel street, 4, 614990, Perm, Russia

**Keywords:** carbazole, chalcone, electrochemical oxidation, pyrimidine, solvatochromism

## Abstract

In this paper we present a synthetic approach to six new D–π–A–D conjugated chromophores containing the *N*-[ω-(4-methoxyphenoxy)alkyl]carbazole fragment. Such readily functionalizable heterocycle as carbazole was used as a main starting compound for their preparation. The investigation of the optical properties has shown that the positive solvatochromism is inherent to the chromophores containing an electron-withdrawing prop-2-en-1-one fragment, while the compounds containing a 2-aminopyrimidine moiety exhibit both positive and negative solvatochromism. The fluorescence quantum yields were experimentally determined for some of the synthesized chromophores; e.g., 1-(5-arylthiophen-2-yl)ethanones quantum yields were found to lie in an interval of 60–80%. Electrochemical oxidation of the synthesized chromophores has resulted in the formation of colored thin oligomeric films that became possible due to the presence of carbazole or pyrrole fragments with free electron-rich positions.

## Introduction

Nowadays a great number of research projects concerning the synthesis of organic conjugated systems with a wide scope of their applications, primarily as materials for organic electronic devices such as light-emitting diodes, field-effect transistors, and electrochromic devices exist. As it is said in a White Paper of Chemical Sciences and Society Summit (CS3) “these materials hold tremendous promise to expand our electronic landscape in ways that will radically change the way society interacts with technology” [1]. Analysis of the results obtained in this area of scientific and technical research confirms that at present time it is really possible to perform tailor-made syntheses of monomers to gain oligomers and polymers with desired properties and characteristics. The conjugated systems including carbazole units possess important advantages when compared with other conjugated aromatic systems [2–4].

First of all, 9*H*-carbazole is a relatively cheap starting material. At the same time, its fully aromatic system renders this heterocycle a good chemical and environmental stability. Nucleophilic substitution of a 9*H* atom proceeds quite smoothly; this feature gives the opportunity to improve solubility and to tune optical, electrochemical, and photovoltaic properties of carbazole-containing compounds. The carbazole moiety has a rigid planar conjugated structure and, as a result, its derivatives are characterized by a high charge mobility. Carbazole undergoes electrophilic substitution mainly at C3 or C6 positions to give 3(6)-substituted derivatives. Nevertheless, nowadays some new synthetic procedures [3,5–7] have given the opportunity to prepare conjugated systems which include 1,8-, 2,7- or 9(3)-carbazolylene units. The incorporation of such moieties changes the optical and electrochemical properties of the resulted conjugated systems [5]. Another effective way to change the properties of a conjugated system involves the modification of substituents attached to a nitrogen atom of the carbazole unit [8]. The long-chain alkyl groups terminated with an aromatic moiety (substituted or non-substituted benzenes, carbazoles, biphenyls, etc.) can be used for these purposes [8–9]. The incorporation of bulky side chains is applied in order to exclude the possible interaction of polymer chains with each other. Such interaction often causes the formation of excimers, which degrades the quality of the blue luminescence. On the other hand, the interaction of bulky side chains with each other can intensify an undesirable charge transfer in thin polymer films that can be overcome by introducing of terminal aromatic moieties into the structure of side chains [10].

In this paper we describe a synthetic approach to some new D–π–A–D conjugated systems which include 9-[ω-(4-methoxyphenoxy)alkyl]carbazole as a donor fragment (D) and prop-2-enone and pyrimidine units as electron acceptor moieties (A). The optical and electrochemical properties of the prepared compounds were investigated by UV–vis absorption spectroscopy and cyclic voltammetry. Electrochemical oxidation of these heterocycles resulted in the formation of thin films on the surface of an ITO working electrode. The morphology of grown films has been investigated by means of scanning tunneling microscopy.

## Results and Discussion

### Synthetic toolbox

The objective of the presented work involves the design and further synthesis of 4,6-diaryl-substituted 2-(pyrrol-1-yl)pyrimidines, embedding 9-[ω-(4-methoxyphenoxy)alkyl]carbazole moieties as substituents, one of which is directly linked with a central pyrimidine core and another one is linked by a rigid thiophene cycle as a π-conjugated bridge. We have applied a usual synthetic protocol which includes eight successive steps: *O*-alkylation of 4-methoxyphenol (1), *N*-alkylation of carbazole (2), formylation or acetylation of a resulted *N*-alkyl carbazole (3), two-staged incorporation of thiophene moiety into obtained methyl ketone (4,5), condensation of the prepared aldehyde and methylketone (6), cyclization of thus prepared chalcone into 4,6-disubstituted 2-aminopyrimidine (7) and preparation of a target product – 4.6-disubstituted 2-(pyrrol-1-yl)pyrimidine via Clausson–Kaas condensation (8).

**Scheme 1:**
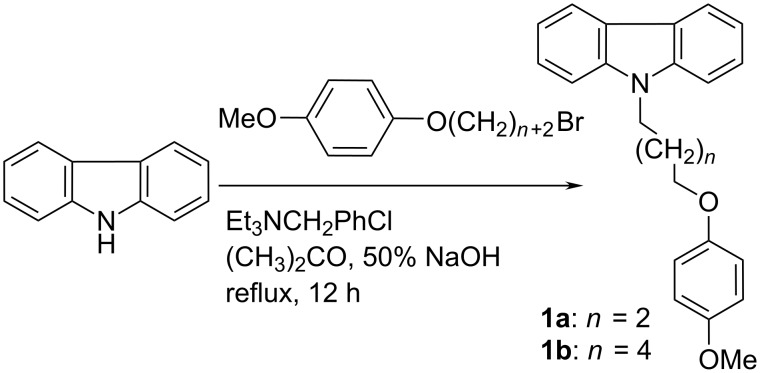
Synthesis of 9-[ω-(methoxyphenoxy)alkyl]-9*H*-carbazoles **1a**,**b**.

Alkylation of 4-methoxyphenol was realized with the help of the traditional base-catalyzed *O*-alkylation process in acetone media [11]. *N*-Alkylated carbazole derivatives can be obtained by different methods such as Mitsunobu reaction [12], Ullmann coupling [13–14], Buchwald–Hartwig amination [15] or *N*-alkylation of carbazole in the presence of alkali metal carbonates under MW irradiation [16]. In our case, the *N*-arylation was realized under phase transfer conditions using triethylbenzylammonium chloride (TEBA) as a catalyst [17–18] (Scheme 1).

**Scheme 2:**
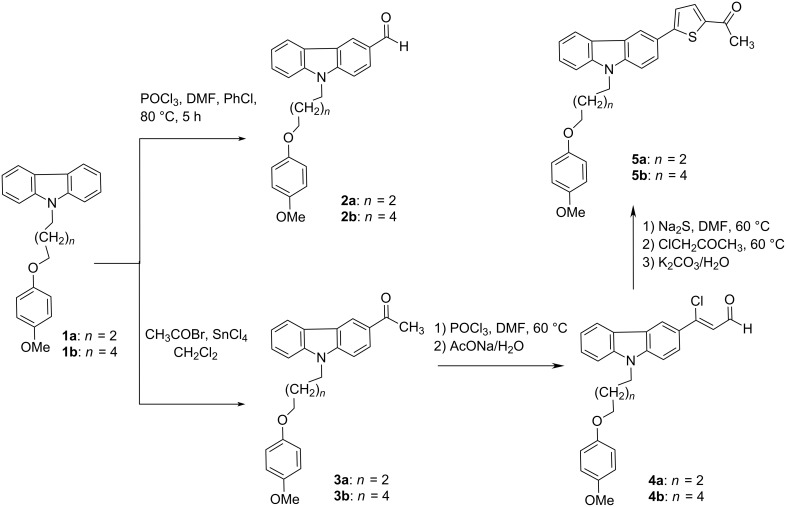
Synthesis of 9-[ω-(4-methoxyphenoxy)alkyl]-9H-carbazole-3-carbaldehydes **2a**,**b** and 1-(5-arylthiophen-2-yl)ethanones **5a**,**b**.

9-[ω-(4-Methoxyphenoxy)alkyl]-9*H*-carbazole-3-carbaldehydes **2a** and **2b**, the carbonyl components for the future crotonic condensation, were prepared by Vilsmeier–Haack formylation of 9-[ω-(4-methoxyphenoxy)alkyl]-9*H*-carbazoles **1a**,**b** [19] (Scheme 2). 3-Acetyl-9-[ω-(4-methoxyphenoxy)alkyl]-9*H*-carbazoles **3a**,**b** were obtained via acetylation of the same carbazoles **1a**,**b** [20]. The presence of an acetyl group in the resulted compounds **3a** and **3b** gives the opportunity to extend their conjugation chain via incorporation of an thiophene moiety with the help of a Vilsmeier–Haack–Arnold reaction. The reaction of POCl_3_, DMF and methylketones **3a** and **3b** resulted in formation of the corresponding 3-aryl-3-chloroprop-2-enals **4a**,**b**, with subsequent sequential treating with Na_2_S and chloroacetone has given rise to 5-aryl-2-acetylthiophenes **5a**,**b** [21] (Scheme 2).

**Scheme 3:**
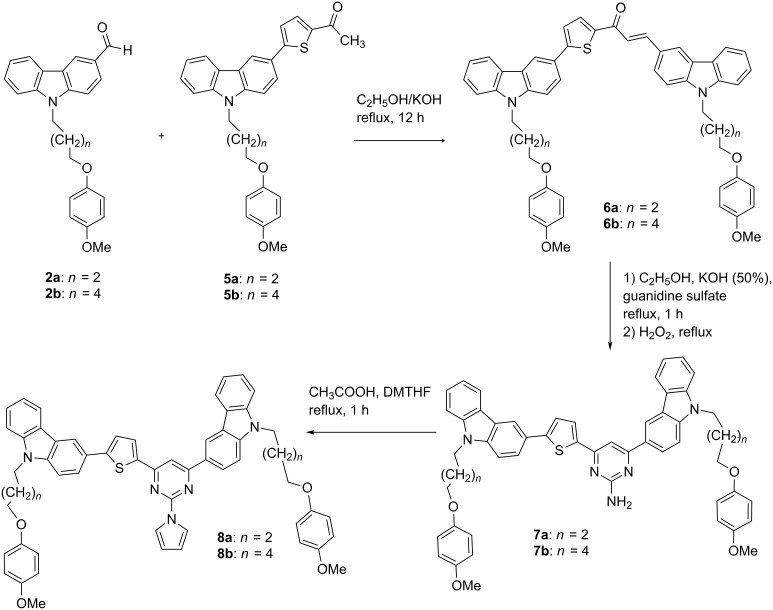
Synthesis of quadrupolar chromophores **6a**,**b**−**8a**,**b**.

Compounds **2** and **5** (Scheme 2) were further used to synthesize a series of D–π–A–D systems (**6**–**8**, Scheme 3), the so-called quadrupolar chromophores, which are known as prospective building blocks for nonlinear optical materials [22]. Crotonic condensation of *N*-substituted carbazole-3-carbaldehydes **2a**,**b** and 1-(5-arylthiophene-2-yl)ethanones **5a**,**b** in the alkaline ethanolic media resulted in the formation of 1,3-diarylsubstituted prop-2-en-1-ones **6a**,**b** [23]. Cyclization of chalcones **6a**,**b** with guanidine sulfate followed by oxidation with hydrogen peroxide gave rise to 2-amino-4,6-disubstituted pyrimidines **7a**,**b** [24]. 2-(1*H*-Pyrrol-1-yl)pyrimidines **8a**,**b** were synthesized via a Clausson–Kaas protocol using 2,5-dimethoxytetrahydrofuran (DMTHF) as a source of succinic aldehyde (Scheme 3) [25]. The purity of the prepared compounds was confirmed by elemental analysis and NMR and IR spectroscopic data.

### Spectroscopic and luminescent properties of the synthesized compounds

The optical properties of all synthesized compounds were studied with the help of UV–vis absorption and fluorescence spectroscopy of their solutions in various solvents. It has been found out that all the prepared compounds bearing 9-[ω-(4-methoxyphenoxy)alkyl]carbazole moieties are readily soluble in dichloromethane (DCM), acetone (DMK), benzene, tetrahydrofuran (THF), dimethylformamide (DMF), dimethyl sulfoxide (DMSO), but are poorly soluble in hot ethanol and not soluble in hexane. On the enlargement of the conjugated system the colour of the compounds solutions in the sunlight expectedly changes from colourless (**2a**,**b**) to orange (**7a**,**b**). Under UV irradiation (λ_eх_ = 315–390 nm) the luminescence of solutions varies from blue (**2a**,**b**) to orange (**7a**,**b**). It has been found out that UV-irradiated solutions of the obtained chromophores **6a**,**b–8a**,**b** change their colours in different solvents of various polarity; for example, compounds **7a**,**b** demonstrate a yellow fluorescence in dichloromethane, DMF, acetone, and an orange one in ethanol, that is most likely connected with the enhancement of the intramolecular charge transfer under UV irradiation.

**Figure 1:**
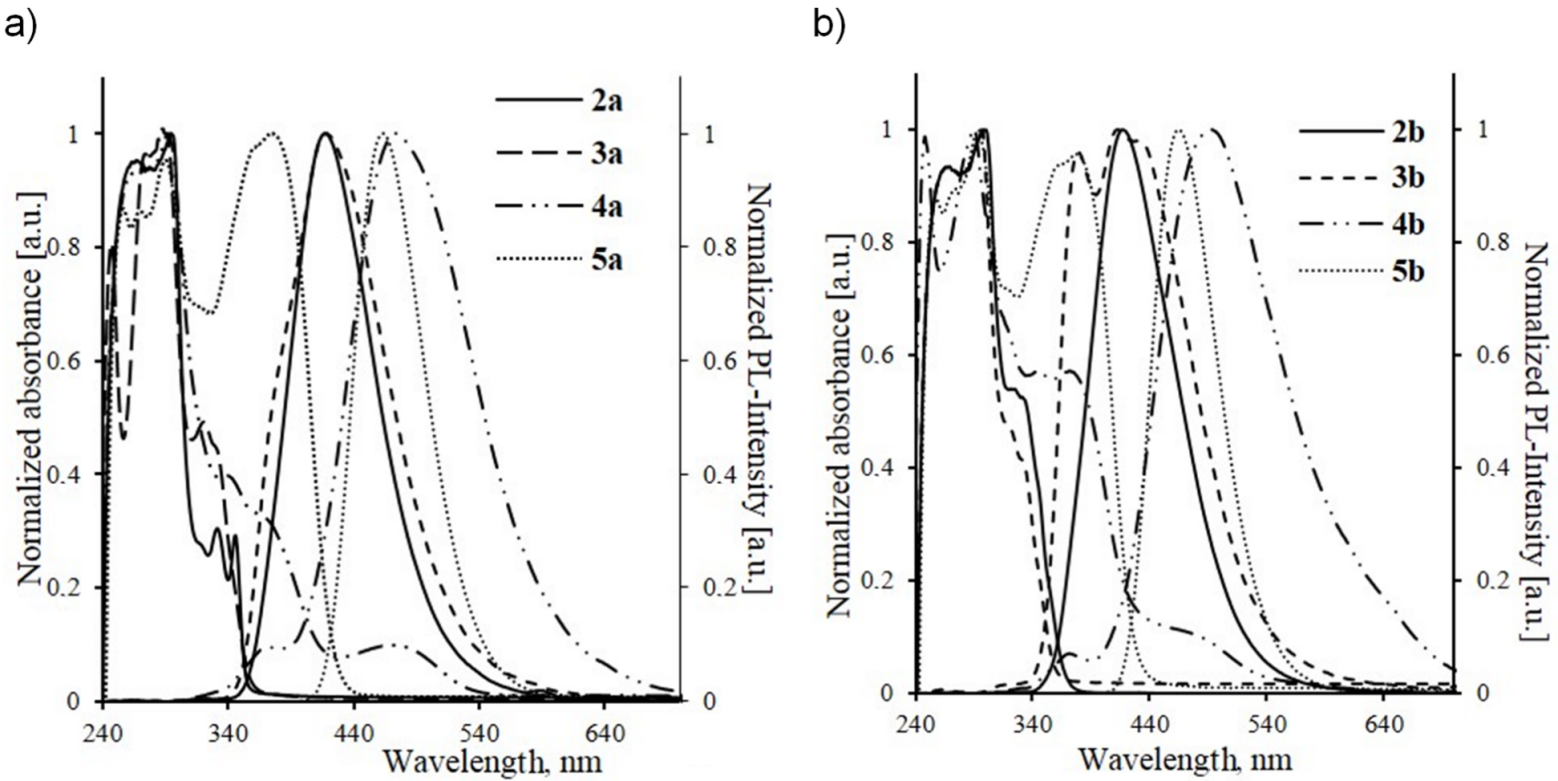
Comparison of UV–vis absorption and fluorescence spectra of compounds **2a**–**5a** (a) and **2b**–**5b** (b) in CHCl_3_ (*c* = 10^−4^ mol L^−1^).

The prepared compounds differ from each other only by the nature of the substituent at the C3 atom of the carbazole cycle: **2a**,**b** – formyl group, **3a**,**b** – acetyl group, **4a**,**b** – 1-chloro-3-oxoprop-1-en-1-yl moiety and **5a**,**b** – 5-acetylthiophen-2-yl unit. That’s why it is interesting to compare their optical properties. So, we have fulfilled the overlapping of the absorption and emission spectra curves shown in Figure 1a,b. The physicochemical characteristics and measurement data of compounds **2a**,**b**–**8a**,**b** are presented in Table 1.

It has been revealed that 9-[ω-(4-methoxyphenoxy)alkyl]-9*H*-carbazole-3-carbaldehydes **2a**,**b** and 3-acetyl-9-[ω-(4-methoxyphenoxy)alkyl]-9*H*-carbazoles **3a**,**b** are characterized by absorption in the 260–300 nm region, which corresponds to the π–π* electron transition, and also by the presence of less intensive absorption maxima in the 320–335 nm region corresponding to the n–π* electron transition (Table 1). The comparison of the absorption spectra of **3a**,**b** and **5a**,**b** has shown that incorporation of a thiophene unit into the structure of compounds **5a**,**b** enforces the intramolecular charge transfer, which, in its turn, causes the decrease in the difference between the energy of the ground and excited states and the appearance of additional absorption bands in the 350–400 nm region corresponding to the π−π* electron transition. The absorption spectra of 3-aryl-3-chloroprop-2-enals **4a**,**b** are of particular interest since there are practically no data concerning optical properties of such structures.

**Table 1:** Physicochemical characteristics of compounds **2a**,**b**−**8a**,**b**.

Compound	Absorption maxima (λ_max_^abs^), nm^a^	Emission maxima (λ_max_^em^), nm^a^	Stokes shift (Δλ), nm	Band gaps width (*E*_g_^opt^), eV^b^	Ф_F_, %^с^

**2a**	267, 295, 322	418	96	3.25	6.3*
**2b**	269, 298, 325	419	94	3.21	1.7*
**3a**	247, 275, 290, 321	420	99	3.11	–
**3b**	271, 298, 321, 334	378, 412, 434	100	3.25	–
**4a**	267, 294, 340, 373, 470	473	100	2.19	–
**4b**	247, 290, 346, 371, 482	492	121	2.03	–
**5a**	257, 271, 291, 375	465	90	2.78	63.0**
**5b**	257, 273, 293, 351, 373	465	88	2.69	79.3**
**6a**	260, 298, 330, 419	505	86	2.55	19.5**
**6b**	258, 298, 336, 406	484	78	2.57	27.1**
**7a**	306, 346, 402, 477, 610	553	97	1.95	−
**7b**	304, 340, 409, 463, 612	555	111	1.94	−
**8a**	306, 351, 405, 521	483	78	1.97	−
**8b**	316, 358, 407, 538	480	73	1.94	−

^a^Absorption and emission spectra were measured for CHCl_3_ solutions (10^−4^ M); ^b^*E*_g_^opt^ was calculated on the basis of the absorption edge value (λ_onset_), *E*_g_^opt^ = 1240/ λ_onset_; ^c^fluorescence quantum yield was determined relative to quinine bisulfate* in 0.1 N H_2_SO_4_ or 3-aminophthalimide** in EtOH as a standard.

The absorption maxima in the spectra of chloropronenals **4a**,**b** located within the 335–370 nm region correspond to the π–π* electron transition arising from the conjugation of the carbonyl group with a C2–C3 double bond and C3 chlorine atom. The broadened absorption maximum in the 460–510 nm region (at the interface between blue and green spectrum regions) characterizes the n–π* electron transition, that occurs most likely due to the presence of a chlorine atom with lone electron pairs.

**Figure 2:**
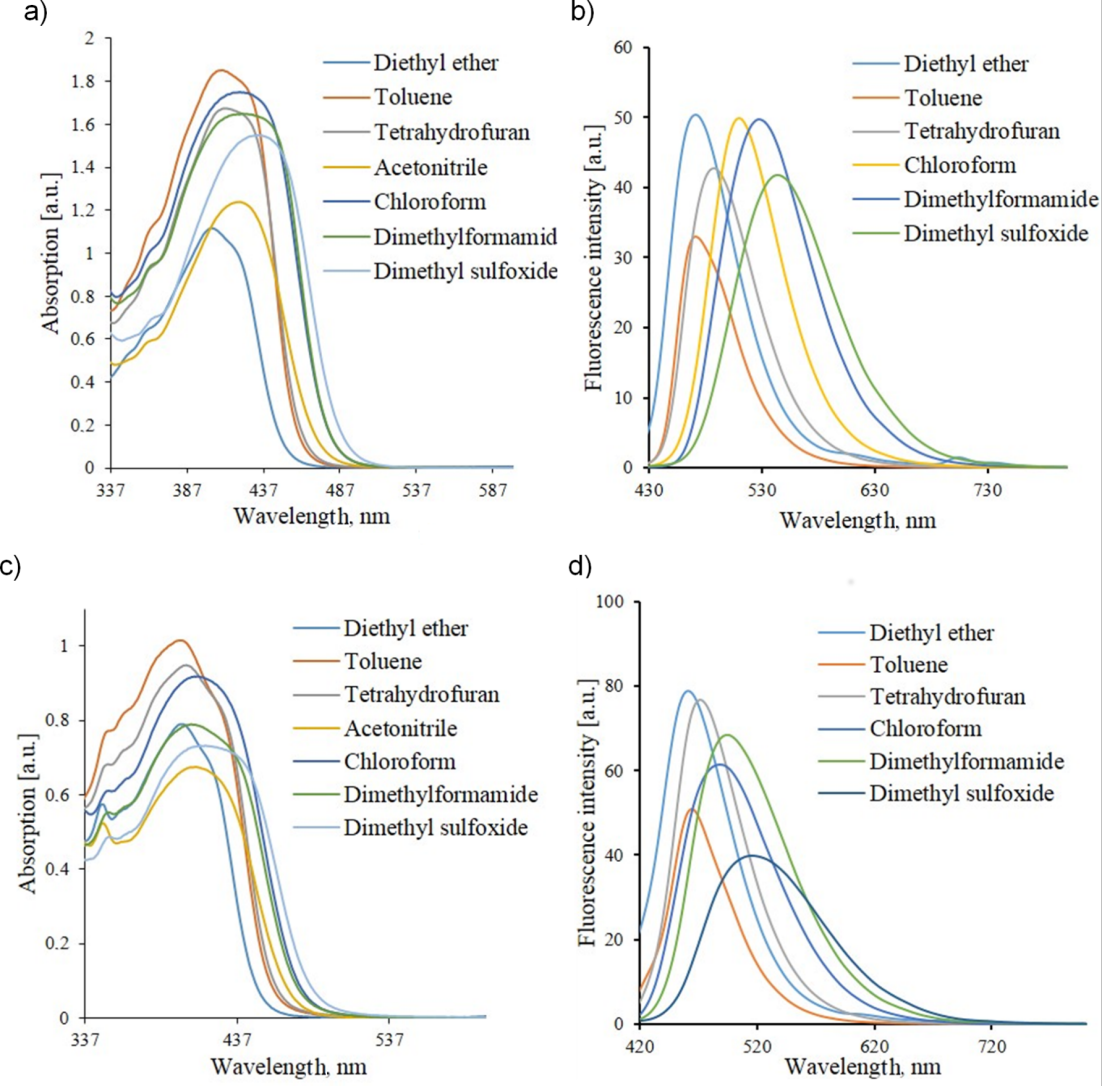
Comparison of UV–vis absorption and fluorescence spectra of compounds **6a** (a, b), **6b** (c, d) in various solvents (*c* = 10^−4^ mol L^−1^).

The above mentioned intramolecular charge transfer inherent to quadrupolar chromophores (D–A–D or A–D–A), also leads to the generation of the positive solvatochromism effects in these systems [26], which exhibit the redshift of a longwave absorption/emission maxima along with the increase of a solvent polarity. Maybe this is a result of a molecule symmetry breaking in its excited state [27]. Taking into account all the factors mentioned above, we have started the investigation of solvatochromic properties of chromophores **6a**,**b** and obtained their absorption and emission spectra in a set of various solvents (diethyl ether, toluene, THF, CHCl_3_, DMF, DMSO). The choice of solvents was made on the basis of a solvatochromic parameter value (π*), which characterizes the ability of solvents to stabilize a neighboring charge or dipole by virtue of nonspecific dielectric interactions [28]. This parameter was determined by Kamlet, Abboud and Taft [29]. The measurement results are displayed in Figure 2.

The obtained data demonstrate that the increase of solvent polarity affords the redshift of the longwave UV absorption maxima and of fluorescence maxima as well, and in the last case this shift is more pronounced. This indicates that the excited state of the molecule is more polar than the ground state, and, therefore, when a solvent is replaced by a more polar one, a large stabilization of the excited state in comparison with the ground state takes place, as evidenced by the quantum-chemical calculations carried out with the help of the Fire Fly package [30–31] on a PGU Tesla supercomputer.

All calculations of the dipole moments, HOMO and LUMO energies were performed on the B3LYP/6-31G* level for the gas phase and for solutions in toluene, chloroform and dimethyl sulfoxide. The solvent parameters were taken into account in the DPCM solvation model [30–31]. All geometries were initially fully optimized for energy in the ground state using the same method. Calculations of the absorption maxima values and the dipole moments in the first excited singlet state were performed on the TD-B3LYP/6-31G* level.

The results, which are presented in Table 2, show that the increase of the solvents polarity has brought about the increase of a molecule dipole moment both in the ground and excited states, and the redshift of an absorption band. The main contribution to the excitation energy of the S1 state in all cases corresponds to the electron transition from the highest occupied molecular orbital (HOMO) onto the lowest unoccupied molecular orbital (LUMO).

**Table 2:** The values of the dipole moments in the ground (μ^0^) and excited (μ*) states, the frontier orbitals energies and the absorption maxima in the gas phase and in various solvents calculated by B3LYP/6-31G* and TD-B3LYP/6-31G* methods for compound **6a**.

	μ^0^, D	*E*(HOMO), eV	*E*(LUMO), eV	μ*, D	λ_max_, nm

gaseous phase	5.98	−5.25	−1.85	19.24	414
toluene	7.47	−5.35	−2.11	21.68	435
CHCl_3_	8.43	−5.39	−2.26	22.90	448
DMSO	9.51	−5.43	−2.42	24.31	466

**Figure 3:**
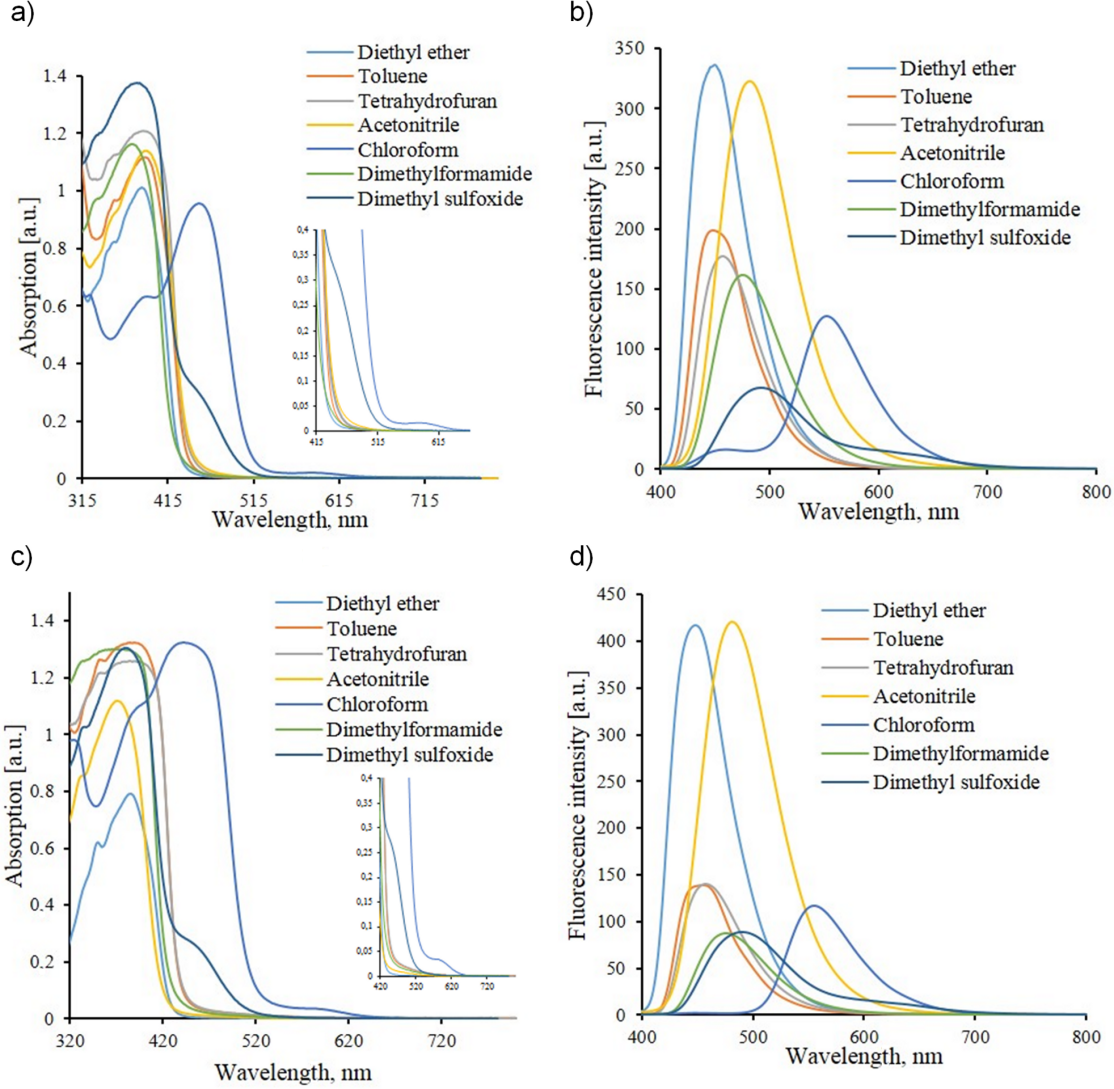
Comparison of UV–vis absorption and fluorescence spectra of compounds **7a** (a, b) and **7b** (c, d) in various solvents (*c* = 10^–4^ mol L^–1^).

We have also investigated the solvatochromic behavior of chromophores **7a**,**b**. An additional absorption maximum was found in the longwave region of the spectra recorded for chloroform and dimethyl sulfoxide solutions of these compounds (Figure 3). The replacement of diethyl ether by chloroform causes a bathochromic shift of the longwave absorption band. Further replacement of the solvents by more polar ones results in hypsochromic shifts.

**Figure 4:**
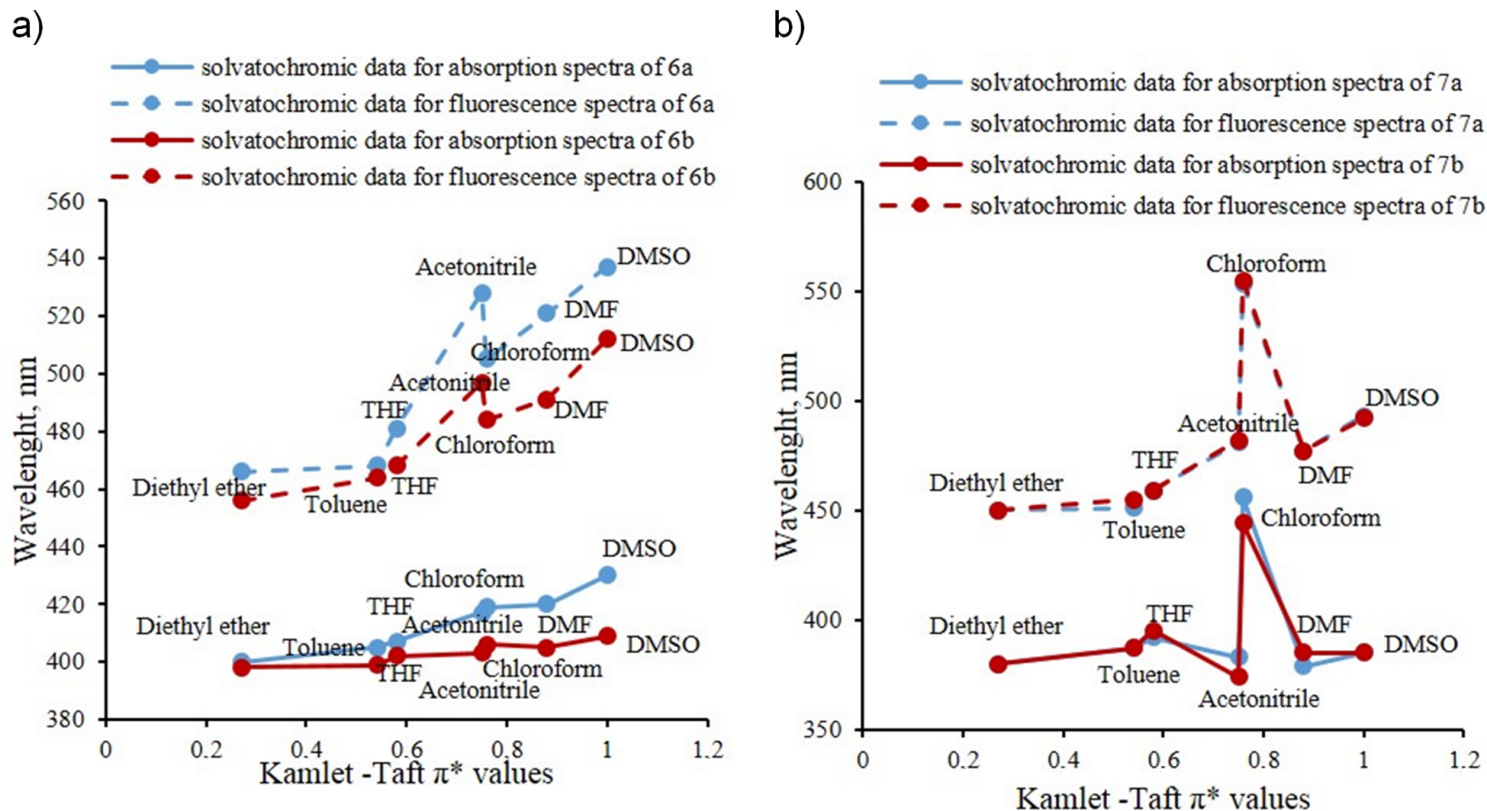
Correlation between Kamlet–Taft π* parameters [29] and the absorption and emission maxima wavelength of chalcones **6a**,**b** (a) and 2-aminopyrimidines **7a**,**b** (b) in the series of solvents.

The correlation between Kamlet–Taft π* parameters [29] and the absorption or emission maxima wavelength is clearly demonstrated by graphs presented in Figure 4. More detailed spectral data of chalcones **6a**,**b** and 2-aminopyrimidines **7a**,**b** are collected in a Table placed in Supporting Information File 1.

**Figure 5:**
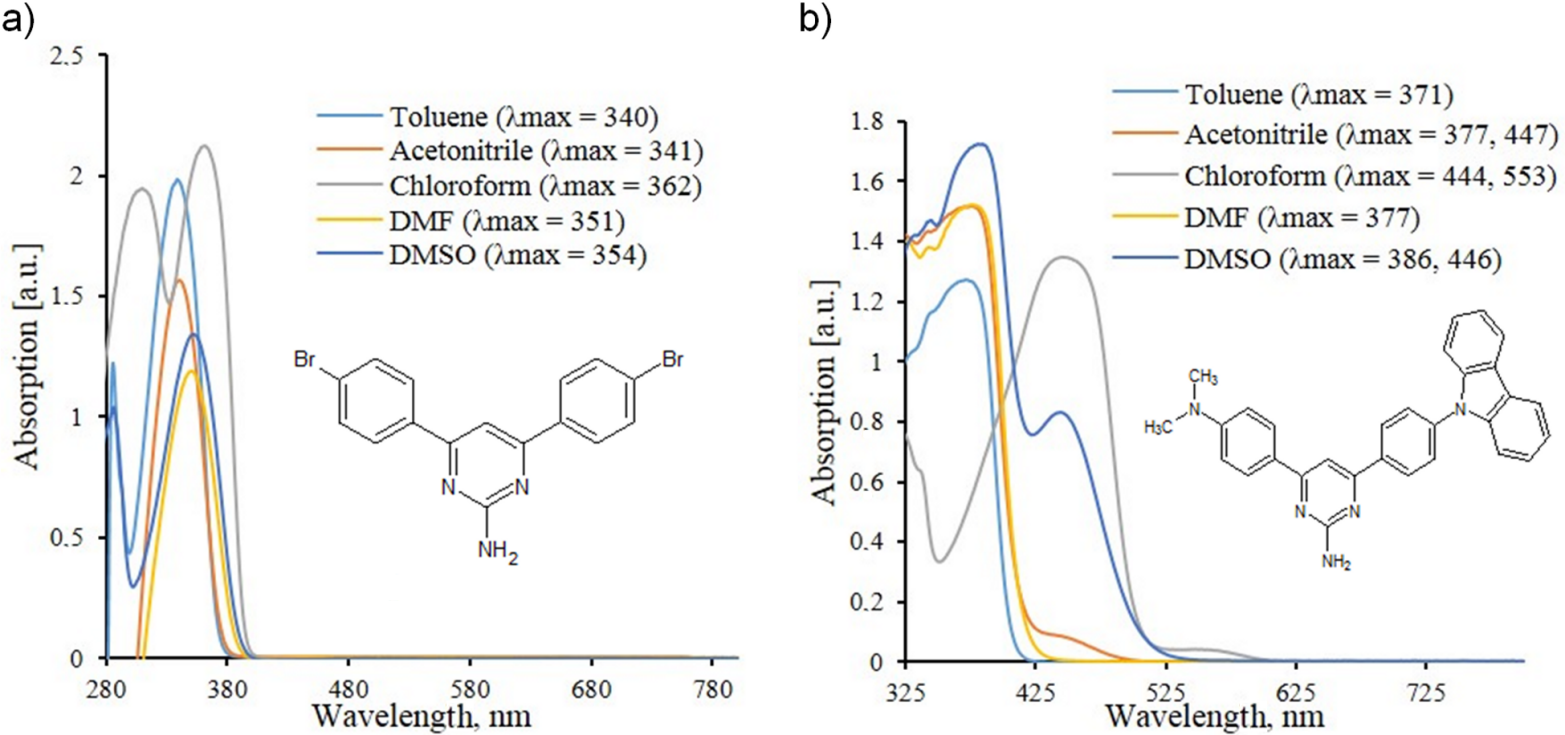
Comparison of UV–vis absorption spectra of 2-amino-4,6-di(4-bromophenyl)pyrimidine and 2-amino-4-[4-(9*H*-carbazol-9-yl)phenyl]-6-[4-(dimethylamino)phenyl]pyrimidine in various solvents (*C* = 10^−4^ mol L^−1^).

Further it has been established that solvatochromism is inherent not only to these newly synthesized chromophores **7a**,**b**, but also to the whole set of 2-amino-4,6-diarylsubstituted pyrimidines even of the less complicated structure. Here we present the results of solvatochromic studies of two previously synthesized 2-aminopyrimidines – symmetrical 2-amino-4,6-di(4-bromophenyl)pyrimidine and asymmetrical 2-amino-4-[4-(9*H*-carbazol-9-yl)phenyl]-6-[4-(dimethylamino)phenyl]pyrimidine. The measurements were done for the same set of solvents; the results are shown in Figure 5.

The replacement of such a nonpolar solvent as toluene by some more polar solvent, chloroform, has resulted in the insignificant bathochromic shift of a longwave absorption maximum in the case of 2-amino-4,6-di(4-bromophenyl)pyrimidine (λ_max_^abs^ = 340 nm for the toluene solution and 362 nm for the chloroform one). In the case of asymmetrical 4-(4-*N*,*N*-dimethylamino)phenyl-6-[4-(9*H*-carbazolyl)phenyl]-2-aminopyrimidine this redshift was more significant ≈70 nm (λ_max_^abs^ = 371 nm for the toluene solution and 444 nm for the chloroform one). This fact can be explained by the presence of electron donating groups in the structure of substituents at C4 and C6 atoms of the pyrimidine core. At the same time, it is possible the protons of the NH_2_ group of the pyrimidine core interact with the *N*,*N*-dimethylamino group of another molecule, thus inducing the rearrangement of electronic density of both molecules [32]. The further increase in the solvent polarity leads in both cases to a hypsochromic shift of a long-wave absorption maximum.

**Figure 6:**
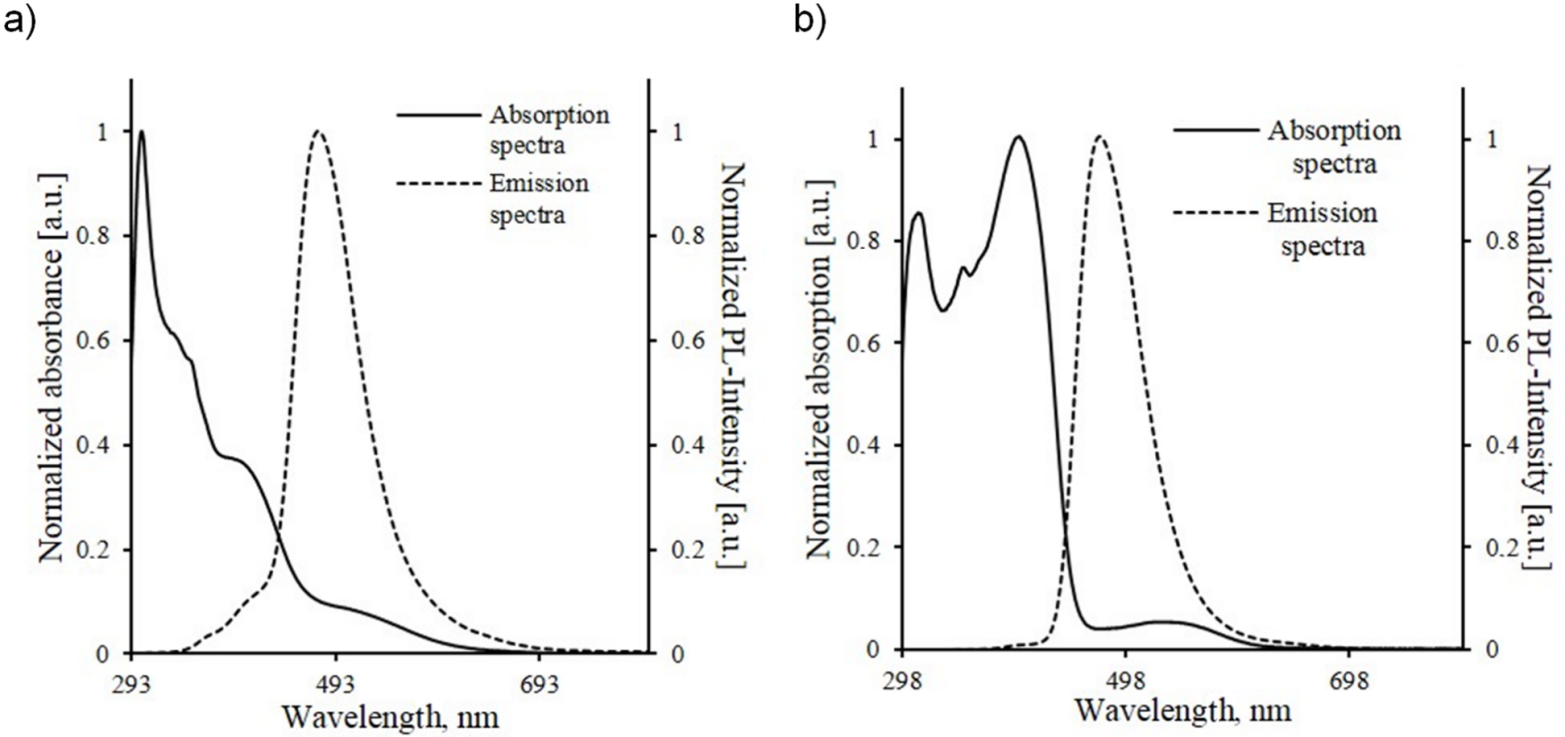
UV–vis absorption and fluorescence spectra of compounds **8a** (a), **8b** ( b) in CHCl_3_ (*c* = 10^−4^ mol L^−1^).

Absorption and emission spectra of 2-(1*H*-pyrrol-1-yl)pyrimidines **8a**,**b** are presented in Figure 6. Absorption bands in the 280–450 nm region characterizing π–π* electron transitions and a low-intensity absorption maxima (at 535 nm for **8a** and at 540 nm for **8b**) which corresponds to the effective intramolecular charge transfer, are typical for this series of compounds.

### Electrochemical properties of the synthesized compounds

**Figure 7:**
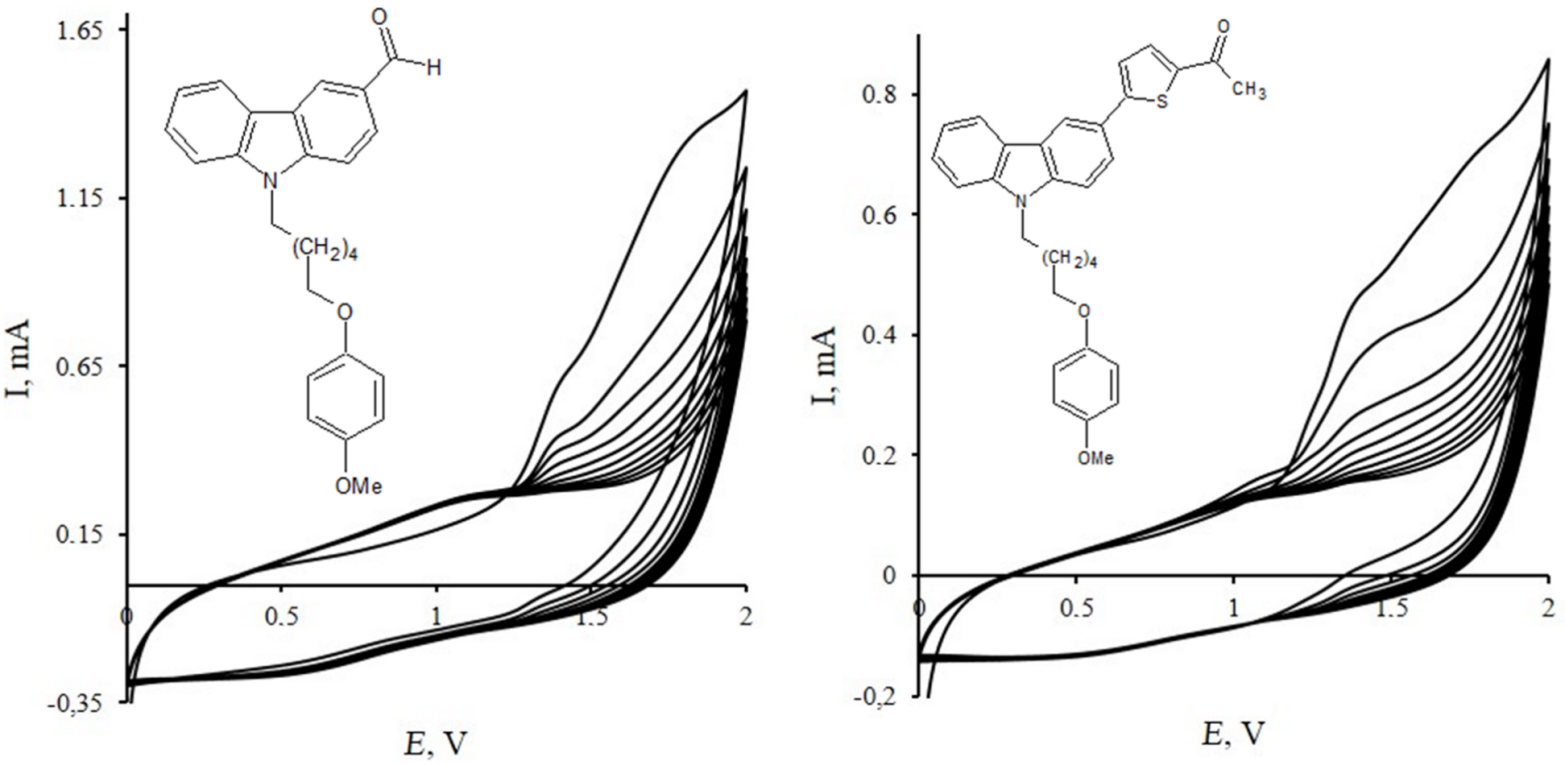
Cyclic voltammograms of compounds **2b** (a), **5b** (b); WE – carbon-pyroceramic electrode, 10 cycles, Et_4_NClO_4_, V_scan_ 50 mV∙s^−1^, CH_3_CN/CH_2_Cl_2_ (9:1, v/v).

**Figure 8:**
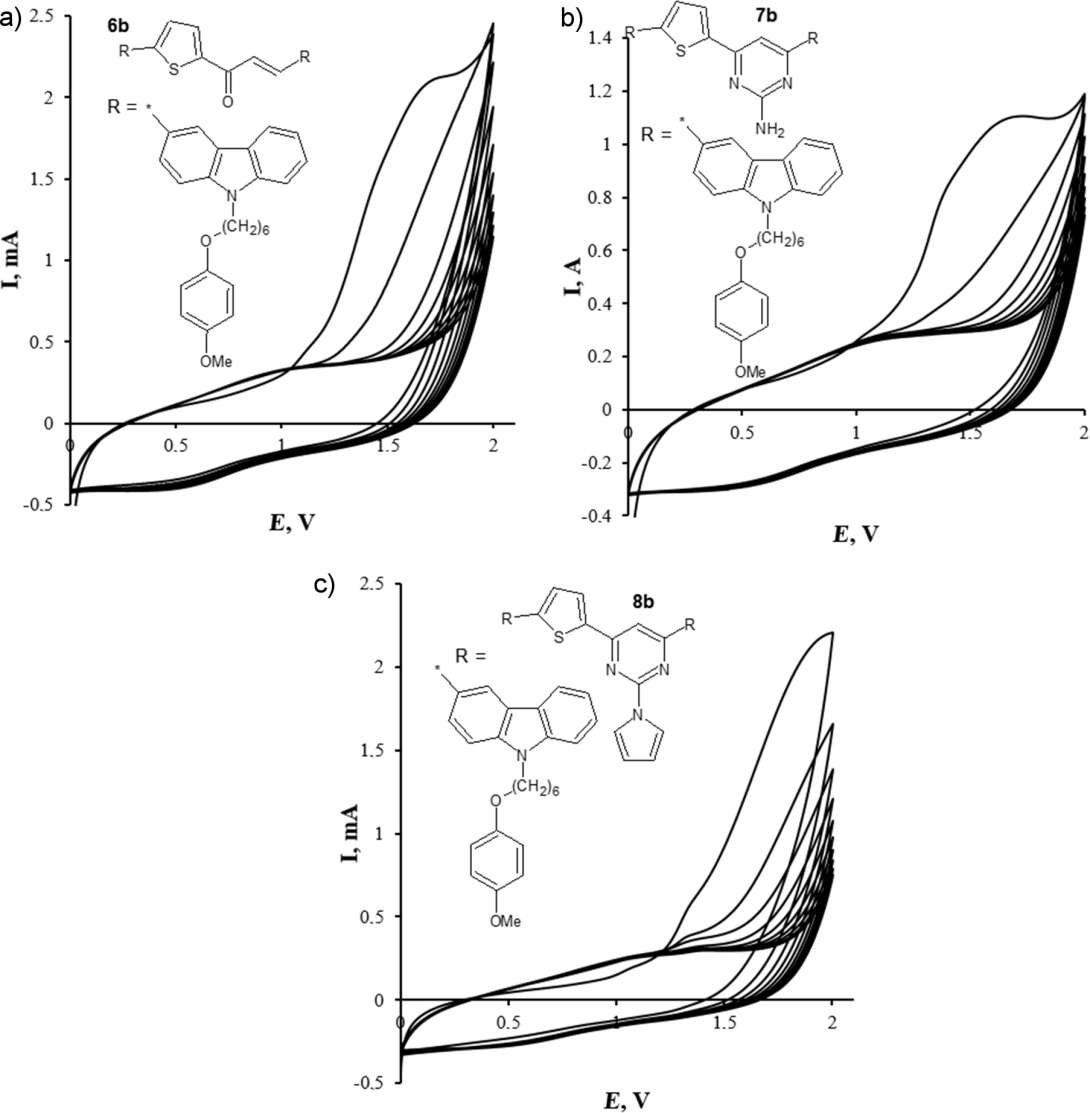
Cyclic voltammograms of compounds **6b** (a), **7b** (b), **8b** (с); WE – carbon-pyroceramic electrode, 10 cycles, Et_4_NClO_4_, V_scan_ 50 mV∙s^−1^, CH_3_CN/CH_2_Cl_2_ (9:1, v/v).

The electrochemical behavior of the synthesized chromophores, each containing an electroactive carbazole unit in their structure, is of special interest. The presence of free C3 and C6 positions in this heterocyclic moiety makes it possible to form bicarbazyl structures as a result of electrochemical oxidation, which mechanism has been described in detail by K. Karon et al. [33]. Electrochemical properties of the obtained compounds were investigated by cyclic voltammetry (CV). The measurements were carried out for MeCN/CH_2_Cl_2_ (9:1) solutions containing Et_4_NClO_4_ (*c* = 10^−3^ mol L^−1^) as a supporting electrolyte in a three-electrode cell (RE – Ag|AgCl; SE – Pt wire; WE – carbon-pyroceramic disk or an ITO-covered glass plate). The redox potentials are referenced to the ferrocene/ferrocenium couple (Fc/Fc^+^). The data are presented in Figure 7 and Figure 8 and summarized in Table 3. The first cycle of the 9-[ω-(4-methoxyphenoxy)alkyl]-9*H*-carbazole-3-carbaldehydes **2a**,**b** CV curves has shown two oxidation waves with potential peak values at 1.39 V (*E*_ox_^1^) and 1.81 V (*E*_ox_^2^) (Figure 7a). These peaks correspond to the formation of cation radical (*E*_ox_^1^) and dication (*E*_ox_^2^); the last one loses two protons and forms a dimer structure, which is depicted in the layout of a cathodic speck of a CV curve (*E*_red_^1^ = 1.08 V, *E*_red_^2^ = 1.35 V). The second and subsequent cycles are characterized by the presence of two reversible peaks (*E*_ox_^1^/*E*_red_^1^ = 1.07/0.64 V, *E*_ox_^2^/*E*_red_^2^ = 1.35/1.26 V), which correspond to the formation of cation radical (*E*_ox_^1^) and dication (*E*_ox_^2^) of a generated dimer. A similar picture was also observed for oxidation processes taking place in the solutions of 1-({5-{9-[6-(4-methoxyphenoxy)hexyl]-9*H*-carbazolyl}thiophen-2-yl})ethanone (**5b**) and **2b** (Figure 7).

The CV curves of the first potential scan obtained in the process of electrochemical oxidation of chromophores **6**–**8** are characterized by lower values of the oxidation potentials if compared with those of compounds **2a**,**b** and **5a**,**b**: *E*_ox_^1^ = 1.12 V, *E*_ox_^2^ = 1.68 V (**6b**); *E*_ox_^1^ = 1.06 V, *E*_ox_^2^ = 1.65 V (**7b**), *E*_ox_^1^ = 1.05 V, *E*_ox_^2^ = 1.34 V (**8b**). This fact can be explained by elongation of a conjugation chain in heterocycles **6** and **8**. Further potential sweep demonstrates a broadened oxidation peak (**6b**: *E*_ox_^1^ = 0.91 V, **7b**: *E*_ox_^1^ = 0.95 V, **8b**: *E*_ox_^1^ = 1.04 V), which represents the formation of a stable dication of a 6,6’-bicarbazyl structure (Figure 8 and Table 3).

**Table 3:** Electrochemical characteristics of compounds **6**–**8** versus Fc/Fc+.

Compounds	**6a**	**6b**	**7b**	**8a**	**8b**

oxidation potential (*E*_ox_), V^a^	1.11, 1.61	1.12, 1.68	1.06, 1.65	1.06, 1.32, 1.67	1.05, 1.34
reduction potential (*E*_red_), V^a^	0.60	0.59	0.56	0.58	0.62
oxidation onset potential (*E*_ox_^onset^), V^a^	1.10	1.06	1.10	1.12	1.10
reduction onset potential (*E*_red_^onset^), V^a^	−0.98	−1.04	0.85	−0.84	−0.83
*E*_HOMO_, eV^b^	−5.57	−5.53	−5.57	−5.59	−5.57
*E*_LUMO_, eV^b^	−3.49	−3.43	−3.62	−3.63	−3.64
*E*_g_^el^*,* eV^c^	2.08	2.1	1.95	1.96	1.93

^a^The electrochemical measurements were carried for monomer solution (*c* = 10^−3^ M) in a CH_3_CN/CH_2_Cl_2_ (9:1) mixture at room temperature with Et_4_N^+^ClO_4_^−^ as a background electrolyte (*c* = 0.1 mol/L) («Potentiostat/Galvanostat/ZRA Interface 1000» and three electrode electrochemical cell with Si(C)-disk, carbon-pyroceramic electrode as working electrodes, Pt wire as auxiliary electrode, silver chloride electrode as reference electrode); ^b^*E*_HOMO_ = −(*E*_ox_^onset^_ versus Ag/AgCl_ − *E*_Fc versus AgCl_ + 4.8) eV; *E*_LUMO_ = −(*E*_red_^onset^_ versus Ag/AgCl_ − *E*_Fc versus AgCl_ + 4.8) eV; ^c^*E*_g_^el^ = (*E*_HOMO_ − *E*_LUMO)_ eV.

In addition to these studies, we have executed the electrochemical oxidation of compounds **6–8b** using ITO covered glass plate as a working electrode. These processes resulted in the formation of thin films on the surface of the working electrode. Cyclic voltammograms, film images and the study of the morphology of their surface are presented in detail in Supporting Information File 1.

## Conclusion

To summarize, we have developed an efficient synthetic approach to a family of highly luminescent D–π–A–D conjugated chromophores each containing the flexible *N*-[ω-(4-methoxyphenoxy)alkyl]carbazole fragment. It has been revealed that positive solvatochromism is inherent to the chromophores of the chalcone group, whereas chromophores of 2-aminopyrimidine group exhibit both positive and negative solvatochromism. For some of the compounds, the fluorescence quantum yields were experimentally determined, which values for 1-(5-arylthiophen-2-yl)ethanones series lie in the range of 60–80%. Electrochemical oxidation of these chromophores has given rise to coloured thin oligomeric films, which are formed by the packed in stacks fibers variously oriented toward each other.

## Supporting Information

File 1 Synthetic procedures and characterization data for all compounds **1**–**8** (**a**,**b**).
