# Supplementary material for: New electroactive asymmetrical chalcones and therefrom derived 2-amino- / 2-(1H-pyrrol-1-yl)pyrimidines, containing an N-[ω-(4-methoxyphenoxy)alkyl]carbazole fragment: synthesis, optical and electrochemical properties
Source: Beilstein J Org Chem. 2017 Aug 10;13:1583–95. doi: 10.3762/bjoc.13.158 (PMC5564266; doi:10.3762/bjoc.13.158)

Supporting Information

for

New electroactive asymmetrical chalcones and therefrom derived 2-amino- / 2-(1*H*-pyrrol-1-yl)pyrimidines, containing an *N*-[ω-(4-methoxyphenoxy)alkyl]carbazole fragment: synthesis, optical and electrochemical properties

Daria G. Selivanova[1], Alexei A. Gorbunov[1], Olga A. Mayorova[1], Alexander N. Vasyanin[2], Igor V. Lunegov[3], Elena V. Shklyaeva[4,5], Georgii G. Abashev[*1,4]

Address: [1]Laboratory of Active Reagents Synthesis, Institute of Technical Chemistry, Russian Academy of Sciences, Ural Division, Academician Korolev street, 3, 614990, Perm, Russia, [2]Department of Analytical Chemistry, Perm State University, Bukirev street, 15, 614990, Perm, Russia, [3]Department of Physics, Radio Electronics and Information Security, Perm State University, Bukirev Street, 15, 614990, Perm, Russia, [4]Department of Organic Chemistry, Perm State University, Bukirev street, 15, 614990, Perm, Russia and [5]Laboratory of organic semiconductors, Natural Sciences Institute, Perm State University, Genkel street, 4, 614990, Perm, Russia

Email: Georgii G. Abashev[*] - gabashev@psu.ru

[*]Corresponding author

Synthetic procedures and characterization data for all compounds 1–8 (a,b)

Content

Experimental S2

General procedure for the synthesis of 9-[ω-(4-methoxyphenoxy)alkyl]-9H-carbazole-3-carbaldehydes (**2a**, **2b**) [2] S3

General procedure for the synthesis of 3-aryl-3-chloroprop-2-enals (**4a**, **4b**) [3]........ S4

General procedure for the synthesis of 1-(5-arylthiophen-2-yl)ethanones (**5a**, **5b**) [3] S5

General procedure for the synthesis of 1,3-diarylsubstituted prop-2-en-1-ones (**6a**, **6b**) [4] S6

General procedure for the synthesis of 2-amino-4R1-6-R2-pyrimidines (**7a**, **7b**) [5] S8
General procedure for the synthesis of 4-R^1-6-R^2-2-(1H-pyrrol-1-yl)pyrimidines (**8a**, **8b**) [6] S9
Spectroscopic and luminescent properties of the synthesized compounds S10
Spectroscopic properties of the thin films and investigation of their fine structure S11
References S16

Experimental

General information

^{1}H NMR spectra (300.05 MHz) were registered on a Varian Mercury plus-300 spectrometer in $CDCl_3$ with hexamethyldisiloxane (0.055 ppm) as an internal standard. The splitting patterns are designated as follows: s (singlet), d (doublet), t (triplet), m (multiplet) and quintet. Signals of a carbazole moiety protons are denoted as Cz, signals of thiophene ring protons as Th. Mass spectra were measured on an Agilent Technologies 6890N/5975B instrument (ionizing electrons energy 70 eV). The elemental analysis was carried out using CHNS-932 LECO Corp analyzer. IR spectra were recorded on a Spectrum Two FTIR spectrophotometer (Perkin Elmer), samples were taken out either in a petroleum jelly or chloroform solutions. UV absorption spectra were measured with Shimadzu UV–vis spectrophotometer UV 2600 (a cell size 10 × 10 × 45 mm). Fluorescence spectra were registered on a Shimadzu RF-5301 PC spectrofluorophotometer: the excitation wavelength – 220 nm, the cell size – 10 × 10 × 45 mm. The reaction course and purity of products were tested by thin-layer chromatography on Sorbfil plates and were visualized with UV light. The mixtures were separated and the target products were purified by column chromatography on silica gel (Lancaster, Silica Gel 60, 0.060–0.2 mm). Electrochemical measurements were carried out at room temperature with a potentiostat/galvanostat ZRA Interface 1000 using a three-electrode cell with a ITO or carbon-pyroceramic electrode working electrode, a Pt wire counter electrode, and a Ag/Ag^+ reference и electrode. The supporting electrolyte was 0.1 M solution of Et_4NClO_4 in the acetonitrile-dichloromethane (9:1) mixture. Potential scan rate was 50 mV/s. The fluorescence quantum yields (Φ_F) were measured with the help of a Shimadzu UV–vis spectrophotometer UV 2600 и Agilent Cary Eclipse Fluorescence

Spectrophotometer EL08083853; the equipment belongs to I. Ya. Postovskii Institute of Organic Synthesis (Ural Branch, Russian Academy of Sciences, Yekaterinburg).

Experimental procedures and analytical data

Compounds **1a,b**, **3a,b** were synthesized according to the procedure published by *Selivanova D.G.et al.*[1].

General procedure for the synthesis of 9-[ω-(4-methoxyphenoxy)alkyl]-9*H*-carbazole-3-carbaldehydes (2a, 2b) [2]

$POCl_3$ (1 ml, 11 mmol) was slowly added at room temperature into a stirred solution of carbazoles **1a/1b** (5 mmol) and DMF (0.5 ml, 7 mmol) in 10 ml of chlorobenzene. The reaction mixture was afterwards stirred for 5 h at 80 °C, then cooled to rt, poured in water (50 ml) and extracted with DCM. The combined extracts were then washed with water, dried ($CaCl_2$) and evaporated. A residue was purified by the column chromatography (silica gel, DCM – as eluent) to give aldehydes **2a**,**b** as white crystal solids, which DCM solutions emit blue fluorescence under ultraviolet exposure.

9-[4-(4-Methoxyphenoxy)butyl]-9H-carbazole-3-carbaldehyde (**2a**): Yield 85% (1.8 g), mp 122−123°C. ^1H NMR (300 MHz, $CDCl_3$, δ, ppm, *J*, Hz): 1.78−1.87 (m, 4H, $NCH_2\underline{CH_2}CH_2CH_2O$), 2.05−2.15 (m, 4H, $NCH_2CH_2\underline{CH_2}CH_2O$), 3.75 (s, 3H, OCH_3), 3.91 (t, ^3J = 6.0, 2H, NCH_2), 4.43 (t, ^3J = 7.2, 2H, OCH_2), 6.77−6.79 (m, 4H, C_6H_4), 7.32 (t, ^3J = 6.9, 1H, Cz), 7.46 (d, ^3J = 7.2, 1H, Cz), 7.51 (d, ^3J = 7.2, 1H, Cz), 7.53 (t, ^3J = 6.9, 1H, Cz), 8.00 (dd, ^3J = 6.9, ^4J = 1.5, 1H, Cz), 8.15 (d, ^3J = 7.8, 1H, Cz), 8.61 (d, ^4J = 1.2, 1H, Cz), 10.09 (s, 1H, CHO). IR (mineral oil), ν, cm^{-1}: 1685 (CHO).

Elemental analysis: calculated for $C_{24}H_{23}NO_3$ (373.44): %C = 77.19, %H = 6.21, %N = 3.75; found: %C = 77.27, %H = 6.19, %N = 3.84.

9-[6-(4-Methoxyphenoxy)hexyl]-9H-carbazole-3-carbaldehyde (**2b**): Yield 80% (1.7 g), mp 79−80°C. ^1H NMR (300 MHz, $CDCl_3$, δ, ppm, *J*, Hz): 1.44−1.53 (m, 4H, $NCH_2CH_2\underline{CH_2CH_2}CH_2CH_2O$), 1.71 (quintet, ^3J = 6.6, 2H, $NCH_2\underline{CH_2}$), 1.92 (quintet, ^3J = 6.9, 2H, $OCH_2\underline{CH_2}$), 3.75 (s, 3H, OCH_3), 3.85 (t, ^3J = 6.3, 2H, NCH_2), 4.35 (t, ^3J = 6.9, 2H, OCH_2), 6.75−6.80 (m, 4H, C_6H_4), 7.31 (t, ^3J = 6.9, 1H, Cz), 7.45 (d, ^3J = 8.1, 1H, Cz), 7.47 (d, ^3J = 8.4,

1H, Cz), 7.52 (t, ^3J = 6.9, 1H, Cz), 7.99 (dd, ^3J = 8.4, ^4J = 1.5, 1H, Cz), 8.15 (d, ^3J = 7.8, 1H, Cz), 8.60 (d, ^4J = 1.5, 1H, Cz), 10.00 (s, 1H, CHO).

IR (mineral oil), ν, cm^{-1}: 1681 (CHO).

Mass spectrum, *m/z* (I_{rel}, %): 402 (15.0) $[M+1]^+$, 401 (54.7) $[M]^+$, 209 (17.8), 208 (100.0) $[M–ArO(CH_2)_5]^+$, 207 (20.4), 180 (35.3) $[ArO(CH_2)_3CH_3]^+$, 152 (11.3) $[ArOCH_2CH_3]^+$, 124 (17.5) $[ArOH]^+$.

Elemental analysis: calculated for $C_{26}H_{27}NO_3$ (373.44): %C = 77.78, %H = 6.78, %N = 3.49; found: %C = 77.85, %H = 6.75, %N = 3.39.

General procedure for the synthesis of 3-aryl-3-chloroprop-2-enals (4a, 4b) [3]

$POCl_3$ (0.9 ml, 10 mmol) was slowly added to 1.1 ml (14 mmol) of DMF at 0 °C; the resulted mixture was then stirred for 10 min at 0 °C and followed by the dropwise addition of 4 mmol of **3a/3b** in 20 ml of DMF. After stirring for 3 h at 60 °C the reaction mixture was cooled down to RT and treated with 10% aqueous solution of sodium acetate to adjust pH 4. A resulted precipitate was filtered off, washed with water, dried in air and used further without additional purification. For characterizing compounds **4a, 4b** were purified by column chromatography (silica gel, DCM). The resulted 3-aryl-3-chloroprop-2-enals are the slowly crystallizing substances of dark red colour. They easily dissolve at room temperature in DCM, DMF, acetone, benzene and in ethanol when heated. Their solutions are yellow with greenish fluorescence under UV exposure.

3-Chloro-3-{9-[4-(4-methoxyphenoxy)butyl]-9H-carbazol-3-yl}prop-2-enal (**4a**): Yield 78% (2 g), ^{1}H NMR (300 MHz, $CDCl_3$, δ, ppm, *J*, Hz): 1.79−1.86 (m, 2H, $NCH_2\underline{CH_2}CH_2CH_2O$), 2.04−2.11 (m, 2H, $NCH_2CH_2\underline{CH_2}CH_2O$), 3.75 (s, 3H, OCH_3), 3.90 (t, ^3J = 6.3, 2H, NCH_2), 4.41 (t, ^3J = 6.9, 2H, OCH_2), 6.78−6.80 (m, 4H, C_6H_4, 1H, C(Cl)=<u>CH</u>), 7.31 (t, ^3J = 6.9, 1H, Cz), 7.43 (d, ^3J = 9, 1H, Cz), 7.48 (d, ^3J = 9, 1H, Cz), 7.52 (t, ^3J = 6.9, 1H, Cz), 7.85 (dd, ^3J = 8.7, ^4J = 1.8, 1H, Cz), 8.13 (d, ^3J = 7.5, 1H, Cz), 8.55 (d, ^4J = 1.5, 1H, Cz), 10.24 (d, ^3J = 6.9, 1H, CHO).

IR ($CHCl_3$), ν, cm^{-1}: 1661 (CHO).

Mass spectrum, *m/z* (I_{rel}, %): 433 (25.5), 312 (32.9), 311 (23.5), 310 (83.4) $[M–ArO]^+$, 292 (22.0), 274 (19.2), 270 (21.7), 269 (16.2), 268 (67.4) $[M–ArO(CH_2)_3]^+$, 247 (12.2), 246 (45.3) $[M–ArOCH_2CH_3–Cl]^+$, 232 (11.7), 218 (14.6), 217 (11.2), 205 (19.3), 204 (100) [M–ArO$(CH_2)_3$–Cl–

$CHO]^+$, 203 (14.0), 192 (12.7), 191 (29.3), 190 (20.6), 176 (20.0), 151 (10.6), 123 (18.5), 109 (15.4), 95 (13.1), 77 (10.5), 55 (13.5).

Elemental analysis: calculated for $C_{26}H_{24}ClNO_3$ (433.93): %C = 71.97, %H = 5.57, %N = 3.23; found: %C = 71.85, %H = 5.61, %N = 3.31.

3-Chloro-3-{9-[6-(4-methoxyphenoxy)hexyl]-9H-carbazol-3-yl}prop-2-enal (**4b**): Yield 86% (1.8 g), ^{1}H NMR (300 MHz, $CDCl_3$, δ, ppm, *J*, Hz): 1.38−1.49 (m, 4H, $NCH_2CH_2\underline{CH_2}\underline{CH_2}CH_2CH_2O$), 1.66−1.73 (m, 2H, $NCH_2\underline{CH_2}$), 1.89−1.93 (m, 2H, $OCH_2\underline{CH_2}$), 3.75 (s, 3H, OCH_3), 3.85 (t, ^3J = 6.3, 2H, NCH_2), 4.32 (t, ^3J = 7.2, 2H, OCH_2), 6.77−6.80 (m, 4H, C_6H_4, 1H, C(Cl)=$\underline{CH}$), 7.30 (t, ^3J = 6.9, 1H, Cz), 7.40 (d, ^3J = 8.7, 1H, Cz), 7.46 (d, ^3J = 7.5, 1H, Cz), 7.51 (t, ^3J = 6.9, 1H, Cz), 7.84 (dd, ^3J = 8.7, ^4J = 1.8, 1H, Cz), 8.13 (d, ^3J = 8.1, 1H, Cz), 8.55 (d, ^4J = 1.8, 1H, Cz), 10.24 (d, ^3J = 6.9, 1H, CHO).

IR ($CHCl_3$), ν, cm^{-1}: 1663 (CHO).

Elemental analysis: calculated for $C_{28}H_{28}ClNO_3$ (461.98): %C = 72.80, %H = 6.11, %N = 3.03; found: %C = 72.91, %H = 6.07, %N = 3.09.

General procedure for the synthesis of 1-(5-arylthiophen-2-yl)ethanones (5a, 5b) [3]

3-Aryl-3-chloroprop-2-enal **4a/4b** (4.5 mmol) was added to a solution of $Na_2S\ 9H_2O$ (1.1 g, 4.5 mmol) in DMF (30 ml). After 3h stirring at 60 °C chloroacetone (0.35 ml, 4.5 mmol) was added and the mixture was then stirred for 2 h more at 60 °C. Potassium carbonate (0.6 g, 4.5 mmol) dissolved in 1 ml of water was added and the reaction mixture was heated at 60 °C for 10 min more, then cooled to rt and poured in water. The resulted precipitate was filtered off, dried in air and purified by column chromatography (silica gel, DCM) to give targeted 1-(5-arylthiophen-2-yl)ethanones **5a** and **5b** as brown crystal solids easily soluble in DCM, acetone, DMF, THF, benzene at room temperature. These solutions are yellow with bright blue-green fluorescence under UV light. Compounds **5a** and **5b** dissolve in hot ethanol to give solutions of yellow colour with green fluorescence under UV light.

1-(5-{9-[4-(4-Methoxyphenoxy)butyl]-9H-carbazol-3-yl}thiophen-2-yl)ethanone (**5a**): Yield 81% (1.7 g), mp 130−131°C. ^{1}H NMR (300 MHz, $CDCl_3$, δ, ppm, *J*, Hz): 1.77−1.87 (m, 2H, $NCH_2\underline{CH_2}CH_2CH_2O$), 2.04−2.11 (m, 2H, $NCH_2CH_2\underline{CH_2}CH_2O$), 2.58 (s, 3H, $COCH_3$), 3.75 (s, 3H, OCH_3), 3.90 (t, ^3J = 6.3, 2H, NCH_2), 4.41 (t, ^3J = 6.9, 2H, OCH_2), 6.79 (s, 4H, C_6H_4), 7.27 (t, ^3J =

7.2, 1H, Cz), 7.36 (d, ^3J = 4.2, 1H, Th), 7.41−7.44 (m, 2H, Cz), 7.49 (t, ^3J = 7.8, 1H, Cz), 7.69 (d, ^3J = 3.9, 1H, Th), 7.76 (dd, ^3J = 8.4, ^4J = 1.8, 1H, Cz), 8.12 (d, ^3J = 7.5, 1H, Cz), 8.37 (s, 1H, Cz).

IR (mineral oil), ν, cm^{-1}: 1651 (C=O).

Mass spectrum, *m/z* (I_{rel}, %): 470 (26.4) $[M+1]^+$, 469 (73.3) $[M]^+$, 347 (17.7), 346 (66.0) $[M–ArO]^+$, 305 (25.9), 304 (100.0) $[M–ArO(CH_2)_3]^+$, 303 (13.5), 262 (12.1), 260 (14.4), 204 (22.1), 203 (12.3), 43 (13.6).

Elemental analysis: calculated for $C_{29}H_{27}NO_3S$ (469.59): %C = 74.17, %H = 5.80, %N = 2.98, %S = 6.83; found: %C = 73.05, %H = 5.71, %N = 3.11, %S = 6.71.

1-(5-{9-[6-(4-Methoxyphenoxy)hexyl]-9H-carbazol-3-yl}thiophen-2-yl)ethanone (**5b**): Yield 76% (1.4 g), mp 131−132°C. ^{1}H NMR (300 MHz, $CDCl_3$, δ, ppm, *J*, Hz): 1.46−1.55 (m, 4H, $NCH_2CH_2\underline{CH_2CH_2}CH_2CH_2O$), 1.67−1.74 (m, 2H, $NCH_2\underline{CH_2}$), 1.89−1.94 (m, 2H, $OCH_2\underline{CH_2}$), 2.58 (s, 3H, $COCH_3$), 3.75 (s, 3H, OCH_3), 3.85 (t, ^3J = 6.0, 2H, NCH_2), 4.32 (t, ^3J = 7.2, 2H, OCH_2), 6.75−6.79 (m, 4H, C_6H_4), 7.27 (t, ^3J = 7.5, 1H, Cz), 7.36 (d, ^3J = 3.9, 1H, Th), 7.39−7.42 (m, 2H, Cz), 7.49 (t, ^3J = 6.9, 1H, Cz), 7.69 (d, ^3J = 3.9, 1H, Th), 7.75 (dd, ^3J = 8.4, ^4J = 1.5, 1H, Cz), 8.12 (d, ^3J = 7.8, 1H, Cz), 8.37 (d, ^4J = 1.5, 1H, Cz).

IR (mineral oil), ν, cm^{-1}: 1652 (C=O).

Elemental analysis: calculated for $C_{31}H_{31}NO_3S$ (497.65): %C = 74.82, %H = 6.28, %N = 2.81, %S = 6.44; found: %C = 74.74, %H = 6.20, %N = 3.93, %S = 6.51.

General procedure for the synthesis of 1,3-diarylsubstituted prop-2-en-1-ones (6a, 6b) [4]

An equimolecular mixture of aldehyde **2a/2b** (2 mmol) and ketone **5a/5b** (2 mmol) in 5 % alcohol solution KOH was refluxed for 12 h, then cooled down to rt and poured in water. A resulted precipitate was filtered off, dried in air and purified with column chromatography (silica gel, EA-Hex 1:1) affording desired prop-2-en-1-ones **6a** and **6b** as bright orange crystal solids easily soluble in DCM, acetone, DMF, THF, benzene at room temperature. These solutions are of yellow-green colour with bright green fluorescence under UV light. Compounds **6a, b** dissolve in hot ethanol to give solutions of yellow colour with yellow fluorescence under UV light.

3-{9-[4-(4-Methoxyphenoxy)butyl]-9H-carbazol-3-yl}-1-(5-{9-[4-(4-methoxyphenoxy)butyl]-9H-carbazol-3-yl}thiophen-2-yl)prop-2-en-1-one (**6a**): Yield 70% (1.1 g), mp 105−106°C. ^{1}H NMR

(300 MHz, $CDCl_3$, δ, ppm, *J*, Hz): 1.78−1.87 (m, 4H, $2NCH_2\underline{CH_2}CH_2CH_2O$), 2.04−2.14 (m, 4H, $2NCH_2CH_2\underline{CH_2}CH_2O$), 3.75 (s, 6H, $2OCH_3$), 3.91 (t, ^3J = 6.0, 4H, $2NCH_2$), 4.40 (t, ^3J = 6.6, 4H, $2OCH_2$), 6.77−6.83 (m, 8H, $2C_6H_4$), 7.27−7.30 (m, 2H, Cz), 7.42−7.50 (m, 5H, Cz), 7.45 (d, ^3J = 3.6, 1H, Th), 7.51 (d, ^3J = 15.0, 1H, C(O)$\underline{CH}$=CH), 7.80 (d, ^3J = 8.4, 2H, Cz), 7.91 (d, ^3J = 3.9, 1H, Th), 8.10 (d, ^3J = 15.3, 1H, C(O)CH=$\underline{CH}$), 8.15−8.17 (m, 3H, Cz), 8.39 (d, ^4J = 1.5, 1H, Cz), 8.43 (d, ^4J = 1.8, 1H, Cz).

^{13}C (400 MHz, δ, ppm): 13.55, 22.16, 25.43, 26.57, 29.18, 42.50, 55.26, 67.71, 108.63, 114.28, 115.070, 117.86, 118.24, 119.05, 119.29, 120.16, 120.99, 122.35, 123.02, 123.91, 124.35, 125.67, 125.85, 125.92, 132.16, 140.31, 140.48, 141.42, 143.14, 144.37, 152.56, 153.53, 153.71, 181.23.

IR (mineral oil), ν, cm^{-1}: 1625 (C=O).

Elemental analysis: calculated for $C_{53}H_{48}N_2O_5S$ (825.02): %C = 77.16, %H = 5.86, %N = 3.40, %S = 3.89; found: %C = 77.07, %H = 5.81, %N = 3.52, %S = 3.80.

3-{9-[6-(4-Methoxyphenoxy)hexyl]-9H-carbazol-3-yl}-1-(5-{9-[6-(4-methoxyphenoxy)hexyl]-9H-carbazol-3-yl}thiophen-2-yl)prop-2-en-1-one (**6b**): Yield 72% (1.3 g), mp 100−101 °C. ^1H NMR (300 MHz, $CDCl_3$, δ, ppm, *J*, Hz): 1.44−1.51 (m, 8H, $2NCH_2CH_2\underline{CH_2}\underline{CH_2}CH_2CH_2O$), 1.67−1.74 (m, 4H, $2NCH_2\underline{CH_2}$), 1.87−1.95 (m, 4H, $2OCH_2\underline{CH_2}$), 3.75 (s, 6H, $2OCH_3$), 3.85 (t, ^3J = 6.3, 4H, $2NCH_2$), 4.33 (t, ^3J = 6.9, 4H, $2OCH_2$), 6.76−6.80 (m, 8H, $2C_6H_4$), 7.27−7.32 (m, 2H, Cz), 7.40−7.49 (m, 5H, Cz), 7.44 (d, ^3J = 3.9, 1H, Th), 7.52 (d, ^3J = 15.6, 1H, C(O)$\underline{CH}$=CH), 7.79 (d, ^3J = 9, 2H, Cz), 7.92 (d, ^3J = 3.9, 1H, Th), 8.10 (d, ^3J = 15.9, 1H, C(O)CH=$\underline{CH}$), 8.13−8.17 (m, 3H, Cz), 8.39 (d, ^4J = 1.5, 1H, Cz), 8.42 (d, ^4J = 1.2, 1H, Cz).

^{13}C (400 MHz, δ, ppm): 14.22, 22.79, 26.08, 27.18, 29.39, 31.73, 43.33, 55.95, 68.56, 109.28, 114.91, 115.68, 118.54, 118.90, 119.65, 119.89, 120.81, 121.65, 123.01, 123.66, 124.56, 124.96, 126.28, 126.48, 126.58, 132.81, 141.04, 141.22, 142.15, 143.81, 145.08, 153.41, 154.02, 154.42, 181.91.

IR (mineral oil), ν, cm^{-1}: 1632 (C=O).

Elemental analysis: calculated for $C_{57}H_{56}N_2O_5S$ (881.13): %C = 77.70, %H = 6.41, %N = 3.18, %S = 3.64; found: %C = 77.61, %H = 6.35, %N = 3.26, %S = 3.73.

General procedure for the synthesis of 2-amino-4R^1-6-R^2-pyrimidines (7a, 7b) [5]

A mixture of 1.5 mmol of an appropriate 1,3-diarylsubstituted prop-2-en-1-one **6a/6b**, a guanidine sulphate (1.3 g, 6 mmol) was refluxed for 1 h in presence of solution KOH (2g) in ethanol (20 ml). Thereafter 0.54 ml of 33% H_2O_2 was slowly added. After refluxing for 2h a resulting reaction mixture was slightly cooled down, poured in ice water. A resulted precipitate was filtered off, dried in air and purified with column chromatography (silica gel, DCM) to give targeted pyrimidines **7a** and **7b** as orange crystal solids easily soluble in common organic solvents with formation solutions of orange colour with bright yellow-orange fluorescence under UV light.

4-{9-[4-(4-Methoxyphenoxy)butyl]-9H-carbazol-3-yl}-6-(5-{9-[4-(4-methoxyphenoxy)butyl]-9H-carbazol-3-yl}thiophen-2-yl)pyrimidine-2-amine (**7a**): Yield 69% (0.9 g), mp 93−94°C. ^{1}H NMR (300 MHz, $CDCl_3$, δ, ppm, *J*, Hz): 1.81−1.87 (m, 4H, $2NCH_2\underline{CH_2}$), 2.07−2.15 (m, 4H, $2OCH_2\underline{CH_2}$), 3.76 (s, 6H, $2OCH_3$), 3.92 (t, ^3J = 6.0, 4H, $2NCH_2$), 4.39−4.47 (m, 4H, $2OCH_2$), 5.22 (s, 2H, NH_2), 6.81 (s, 8H, $2C_6H_4$), 7.30 (t, ^3J = 6.6, 2H, Cz), 7.42−7.49 (m, 6H, Cz), 7.43 (d, ^3J = 3.6, 1H, Th), 7.52 (s, 1H, Pyrimidine), 7.81 (d, ^3J = 8.4, 1H, Cz), 7.85 (d, ^3J = 3.9, 1H, Th), 8.13 (d ^3J = 7.5, 1H, Cz), 8.20 (d, ^3J = 8.4, 1H, Cz), 8.22 (d, ^3J = 7.2, 1H, Cz), 8.43 (s, 1H, Cz), 8.86 (s, 1H, Cz).

Elemental analysis: calculated for $C_{54}H_{49}N_5OS$ (864.06): %C = 75.06, %H = 5.72, %N = 8.11, %S = 3.71; found: %C = 74.95, %H = 5.68, %N = 8.17, %S = 3.82.

4-{9-[6-(4-Methoxyphenoxy)hexyl]-9H-carbazol-3-yl}-6-(5-{9-[6-(4-methoxyphenoxy)hexyl]-9H-carbazol-3-yl}thiophen-2-yl)pyrimidine2-amine (**7b**): Yield 71% (1 g), mp 91−92°C. ^{1}H NMR (300 MHz, $CDCl_3$, δ, ppm, *J*, Hz): 1.46−1.61 (m, 8H, $2NCH_2CH_2\underline{CH_2CH_2}CH_2CH_2O$), 1.66−1.75 (m, 4H, $2NCH_2\underline{CH_2}$), 1.90−1.98 (m, 4H, $2OCH_2\underline{CH_2}$), 3.75 (s, 6H, $2OCH_3$), 3.86 (t, ^3J = 6.3, 4H, $2NCH_2$), 4.31−4.38 (m, 4H, $2OCH_2$), 5.29 (s, 2H, NH_2), 6.78−6.80 (m, 8H, $2C_6H_4$), 7.29 (t, ^3J = 6.6, 2H, Cz), 7.41 (d, ^3J = 4.2, 1H, Th), 7.42−7.49 (m, 6H, Cz), 7.53 (s, 1H, Pyrimidine), 7.80 (dd, ^3J = 8.4, ^4J = 1.8, 1H, Cz), 7.86 (d, ^3J = 3.9, 1H, Th), 8.14 (d, ^3J = 8.1, 1H, Cz), 8.19 (d, ^3J = 8.4, 1H, Cz), 8.22 (d, ^3J = 7.5, 1H, Cz), 8.42 (d, ^4J = 1.5, 1H, Cz), 8.86 (d, ^4J = 1.5, 1H, Cz).

Elemental analysis: calculated for $C_{58}H_{57}N_5O_4S$ (920.17): %C = 75.71, %H = 6.24, %N = 7.61, %S = 3.48; found: %C = 75.60, %H = 6.18, %N = 7.70, %S = 3.56.

General procedure for the synthesis of 4-R^1-6-R^2-2-(1*H*-pyrrol-1-yl)pyrimidines (8a, 8b) [6]

2,5-Dimethoxytetrahydrofuran (0.02 ml, 0.2 mmol) was added to a solution of 2-amino-4R^1-6-R^2-pyrimidine **7a/7b** (0.2 mmol) in 5 ml acetic acid, the resulting mixture was heated under reflux for 1 h (the completion of the reaction was monitored by TLC), then cooled to rt and poured into ice water (20 ml). A resulted precipitate was filtered off, dried in air and purified with column chromatography (silica gel, DCM).

4-{9-[4-(4-Methoxyphenoxy)butyl]-9H-carbazol-3-yl}-2-(1H-pyrrol-1-yl)-6-(5-{9-[4-(4-methoxyphenoxy)butyl]-9H-carbazol-3-yl}thiophen-2-yl)pyrimidine (**8a**): Yield 54% (0.1 g). ^1H NMR (400 MHz, $CDCl_3$, δ, ppm, *J*, Hz): 1.70−1.78 (m, 8H, $2NCH_2CH_2\underline{CH_2}\underline{CH_2}CH_2CH_2O$), 1.88−1.93 (m, 4H, $2NCH_2\underline{CH_2}$), 2.14−2.21 (m, 4H, $2OCH_2\underline{CH_2}$), 3.79 (s, 6H, $2OCH_3$), 3.97 (t, ^3J = 6.3, 4H, $2NCH_2$), 4.47 (t, ^3J = 6.6, 4H, $2OCH_2$), 6.43−6.45 (m, 2H, Pyrrole), 6.81−6.85 (m, 8H, $2C_6H_4$), 7.32−7.37 (m, 2H, Cz), 7.47−7.51 (m, 3H, Cz), 7.51 (d, ^3J = 3.5, 2H, Pyrrole), 7.61 (d, ^3J = 7.8, 1H, Cz), 7.71 (d, ^3J = 7.5, 1H, Cz), 7.89 (d, ^3J = 7.2, 1H, Cz), 7.90 (s, 1H, Pyrimidine), 8.02 (d, ^3J = 3.6, Th), 8.22 (d, ^3J = 8.0, 1H, Cz), 8.30 (d, ^3J = 8.0, 1H, Cz), 8.45 (d, ^3J = 7.6, 1H, Cz), 8.49 (s, 1H, Cz), 8.63 (d, ^3J = 3.6, 1H, Th), 9.04 (s, 1H, Cz), 9.17 (d, ^3J = 8.4, 1H, Cz).

Elemental analysis: calculated for $C_{58}H_{51}N_5O_4S$ (914.12): %C = 76.21, %H = 5.62, %N = 7.66, %S = 3.51; found: %C = 76.13, %H = 5.69, %N = 7.58, %S = 3.58.

4-{9-[6-(4-Methoxyphenoxy)hexyl]-9H-carbazol-3-yl}-2-(1H-pyrrol-1-yl)-6-(5-{9-[6-(4-methoxyphenoxy)hexyl]-9H-carbazol-3-yl}thiophen-2-yl)pyrimidine (**8b**): Yield 52% (0.1 g). ^1H NMR (300 MHz, $CDCl_3$, δ, ppm, *J*, Hz): 1.48−1.53 (m, 8H, $2NCH_2CH_2\underline{CH_2}\underline{CH_2}CH_2CH_2O$), 1.70−1.76 (m, 4H, $2NCH_2\underline{CH_2}$), 1.91−1.98 (m, 4H, $2OCH_2\underline{CH_2}$), 3.74 (s, 3H, OCH_3), 3.75 (s, 3H, OCH_3), 3.87 (t, ^3J = 6.3, 4H, $2NCH_2$), 4.38 (t, ^3J = 6.6, 4H, $2OCH_2$), 6.39−6.40 (m, 2H, Pyrrole), 6.77−6.80 (m, 8H, $2C_6H_4$), 7.28−7.33 (m, 2H, Cz), 7.42−7.53 (m, 3H, Cz), 7.50 (d, ^3J = 3.3, 2H, Pyrrole), 7.57 (d, ^3J = 7.2, 1H, Cz), 7.68 (d, ^3J = 7.2, 1H, Cz), 7.85 (d, ^3J = 8.4, 1H, Cz), 7.88 (s, 1H, Pyrimidine), 7.99 (d, ^3J = 4.2, Th), 8.19 (d, ^3J = 8.1, 1H, Cz), 8.28 (d, ^3J = 7.8, 1H, Cz), 8.43 (d, ^3J = 7.8, 1H, Cz), 8.46 (s, 1H, Cz), 8.60 (d, ^3J = 3.6, 1H, Th), 9.00 (s, 1H, Cz), 9.14 (d, ^3J = 8.1, 1H, Cz).

Elemental analysis: calculated for $C_{62}H_{59}N_5O_4S$ (970.23): %C = 76.75, %H = 6.13, %N = 7.22, %S = 3.30; found: %C = 76.68, %H = 6.19, %N = 7.14, %S = 3.39.

Spectroscopic and luminescent properties of the synthesized compounds

The optical properties of all synthesized compounds were studied with the help of UV–vis absorption and fluorescence spectroscopy. Absorption and photoluminescence (PL) spectra were obtained for their solutions in various solvents. It has been revealed that positive solvatochromism is inherent to the chromophores of the chalcone group, whereas chromophores of 2-aminopyrimidine group exhibit both positive and negative solvatochromism. The measurement results are displayed in Table 1 and Table 2.

Table 1: Solvatochromic data for 1,3-diarylsubstituted prop-2-en-1-ones (**6a,b**) in series of solvents and Kamlet–Taft π* values [7].

Solvents	**π***[a]	**6a**			**6b**		
		λ_{max}^{abs}, nm[b]	λ_{onset}^{abs}, nm[c]	λ_{max}^{em}, nm[d]	λ_{max}^{abs}, nm[b]	λ_{onset}^{abs}, nm[c]	λ_{max}^{em}, nm[d]
Diethyl ether	0.27	400	453	466	398	456	456
Toluene	0.54	405	469	468	399	467	464
Tetrahydrofuran	0.58	407	470	481	402	468	468
Acetonitrile	0.75	417	478	528	403	475	497
Chloroform	0.76	419	487	505	406	482	484
Dimethylformamide	0.88	420	487	521	405	482	491
Dimethyl sulfoxide	1.00	430	496	537	409	493	512

[a]Solvatochromic parameters; [b]absorption maxima; [c]the longest absorption wavelength value; [d]emission maxima.

Table 2: Solvatochromic data for 2-amino 4,6-diarylsubstituted pyrimidines (**7a, 7b**) in series solvents and Kamlet-Taft π*-values [7].

Solvents	**π***[a]	**7a**			**7b**		
		λ_{max}^{abs}, nm[b]	λ_{onset}^{abs}, nm[c]	λ_{max}^{em}, nm[d]	λ_{max}^{abs}, nm[b]	λ_{onset}^{abs}, nm[c]	λ_{max}^{em}, nm[d]
Diethyl ether	0.27	380	440	450	380	437	450
Toluene	0.54	387	445	451	387	450	455
Tetrahydrofuran	0.58	392	448	459	395	454	459
Acetonitrile	0.75	383	451	481	374	440	482
Chloroform	0.76	456, 589	634	553	444, 590	637	555
Dimethylformamide	0.88	379	443	477	385	445	477
Dimethylsulfoxide	1.00	385, 461	519	493, 630	385, 467	510	492, 638

[a]Solvatochromic parameters (The π* scale is an index of solvent dipolarity/polarizability, which measures the ability of the solvent to stabilize a charge or a dipole by virtue of its dielectric effect); [b]absorption maxima; [c]the longest absorption wavelength value; [d]emission maxima.

Spectroscopic properties of the thin films and investigation of their fine structure

Electrochemical oxidation of compounds **6–8b** using ITO electrode as a working one resulted in formation of thin films on its surface. Electrochemical oxidation of 1,3-diaryl substituted prop-2-en-1-one (**6b**) gave rise to a green film, 2-amino-4,6-diarylsubstituted pyrimidine **7b** – to an orange film, 2-(1*H*-pyrrol-1-yl)pyrimidine **8b** – to a brown one (Figure 1).

a)

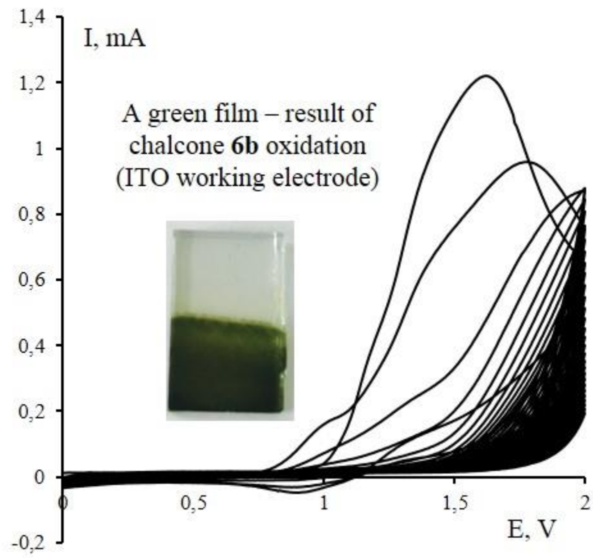

b)

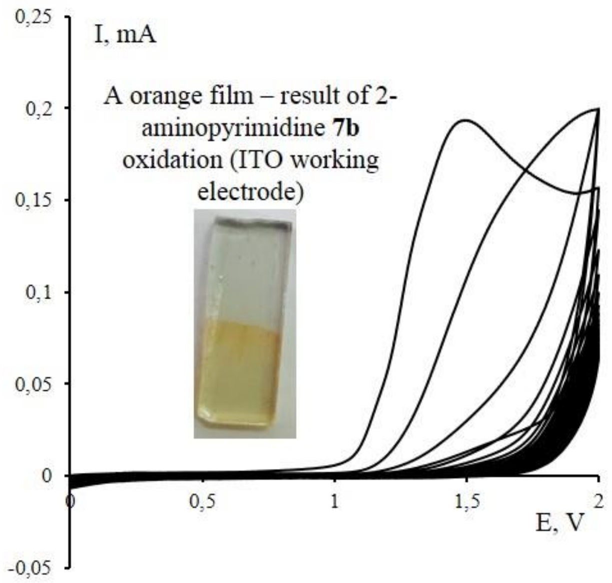

c)

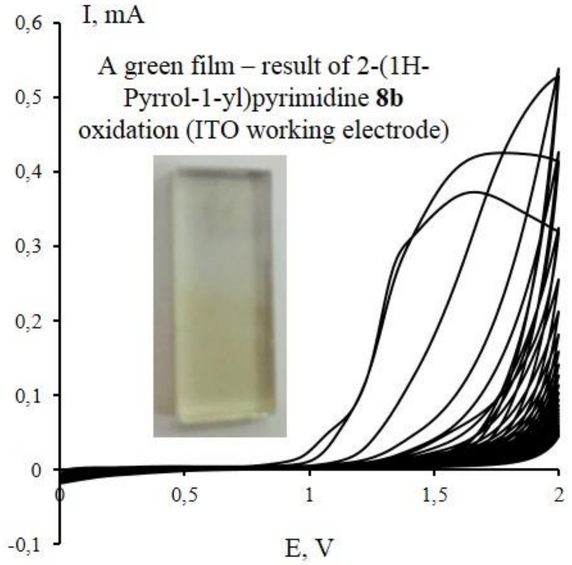

Figure 1: Cyclic voltammograms of compounds **6b** (a), **7b** (b) **8b** (c); WE –ITO-covered plate, 30 cycles, Et_4NClO_4, V_{scan} 50 mV·s^{-1}, CH_3CN:CH_2Cl_2 (9:1, v/v).

I

For the purposes to study spectral characteristics and a surface morphology of all thus prepared films they were carefully washed with distilled water and ethanol, dried in argon-filled sealed glove box Nitrogen Glove Box PlasLabs. The UV–vis absorption spectra of the films are characterized by the presence of a long-wavelength absorption maximum within 600–800 nm

range (Figure 2). The comparative analysis of the obtained spectral data has shown that the replacement of an electron-withdrawing prop-2-en-1-one fragment by 2-amino- or 2- (1*H*-pyrrol-1-yl) pyrimidine units results in a strong bathochromic shift of this long-wavelength absorption maximum at about 110–130 nm.

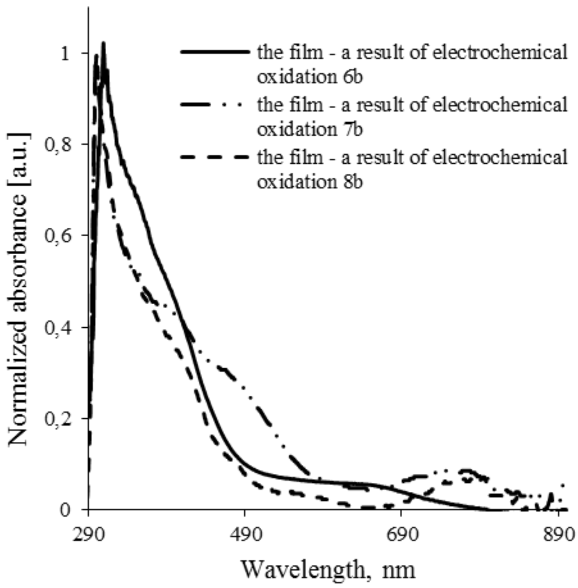

Figure 2: UV–vis absorption spectra of the films obtained by electrochemical oxidation of the compounds **6–8b**.

The morphology of the films surface was studied with the help of scanning tunnel microscopy (Ntegra-Prima). It has been revealed that the film, which were obtained by electrochemical oxidation of chalcone **6b**, has a thickness at about 90 nm and is formed from fibers aggregated in stacks, which are randomly oriented toward each other and toward the surface of the film. The length of these fibers varied in the range from 20 to 70 nm and the thickness – in the 80–100 nm interval (Figure 3a–d).

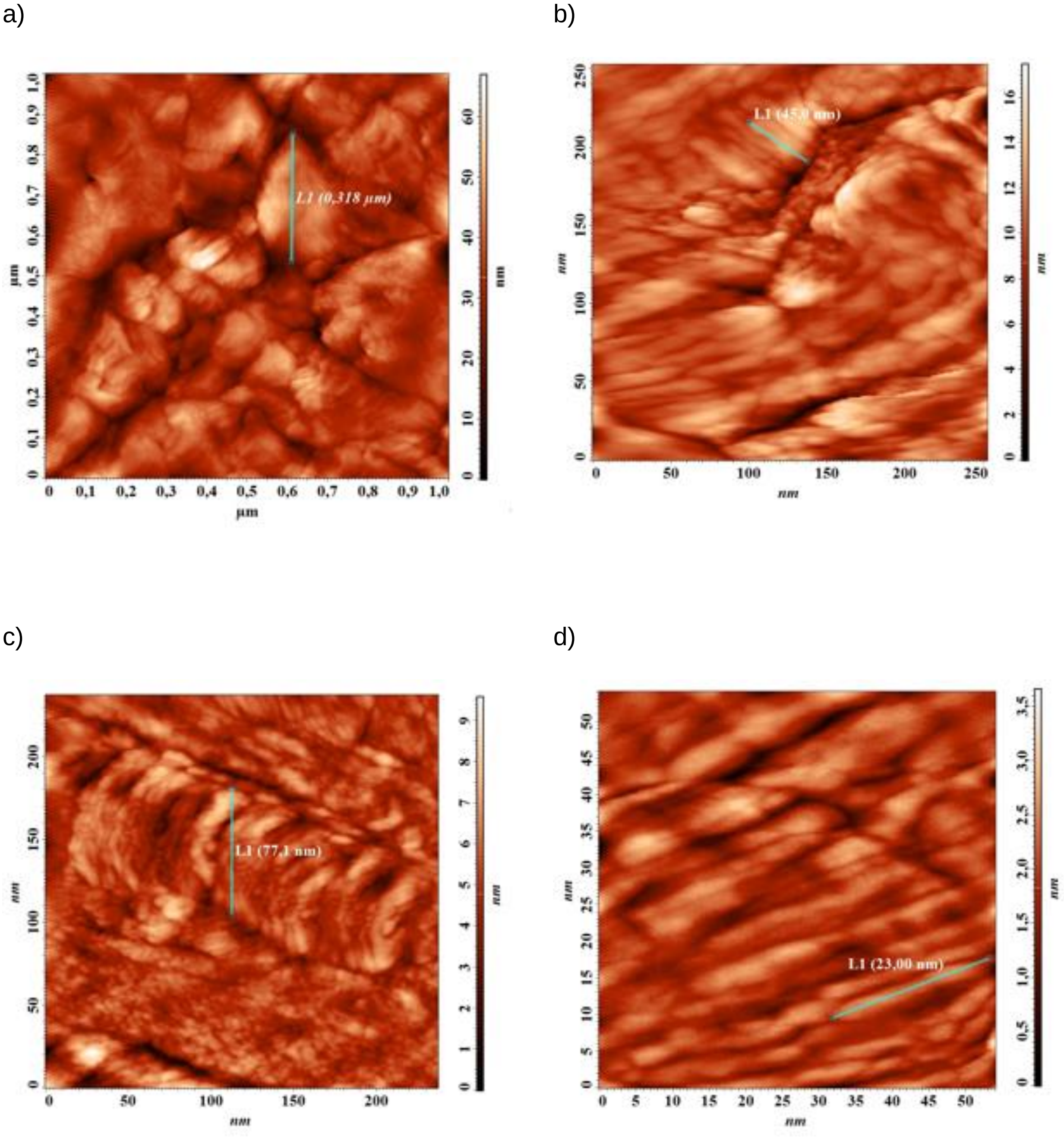

Figure 3: 2D STM images of a chalcone **6b** film of different scan area: 1.0 × 1.0 μm (**a**); 250 × 250 nm (**b**); 200 × 200 nm (**c**); 50 × 50 nm (**d**).

Electrochemical oxidation of 2-amino-4,6-diarylsubstituted pyrimidine **7b** has afforded an orange film of 100 nm thickness. The surface structure of this film is also formed from stacks, which are closely interwoven with each other and make up elongated formations intertwining with each other (Figure 4 a,b).

a) b)

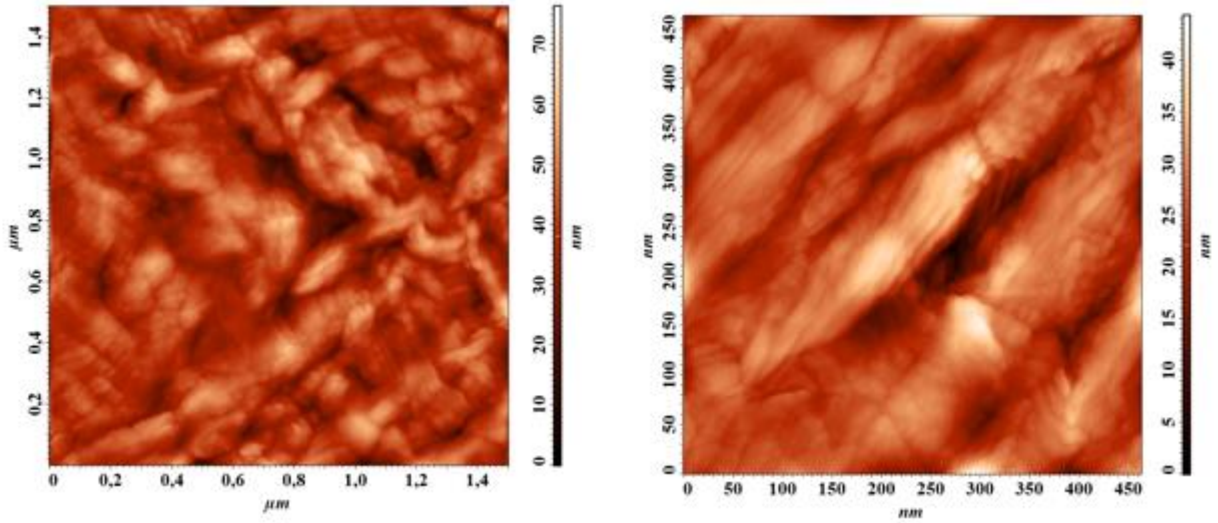

Figure 4: 2D STM images of a 2-aminopyrimidine **7b** film of different scan area: 1.4x1.4 μm (**a**); 450x450 nm (**b**).

The electrochemical oxidation of 2-(1*H*-pyrrol-1-yl)pyrimidine **8b** has resulted in formation of a brown coloured film of 20–25 nm thickness, which structure definitely differs from the structures of the films obtained by electrochemical oxidation of chalcone **6b** and 2-aminopyrimidine **7b**. The structure of this film is formed by 100–200 nm fibers parallel to each other with signs of self-organization effects. When scanning the 160 × 160 nm region, there have been determined the domains of about ≈5 nm in size, which corresponds to the size of the 2-(1*H*-pyrrol-1yl)pyrimidine **8b** molecule (≈40 Å) (Figure 5a,b).

a) b)

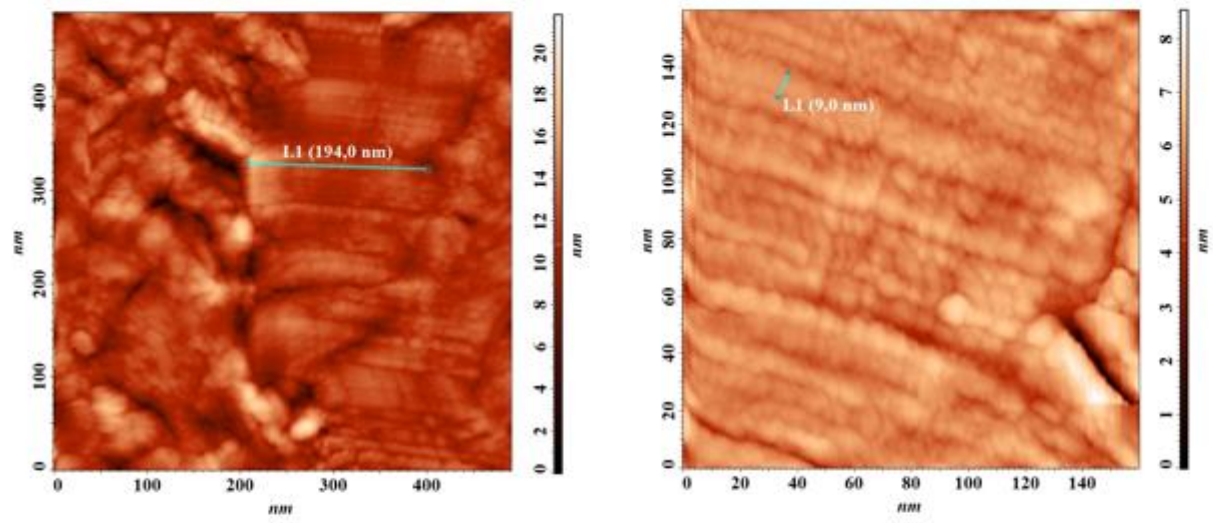

Figure 5: 2D STM images of a 2-(1H-Pyrrol-1-yl)pyrimidine **8b** film of different scan area: 400x400 nm (**a**); 140x140 nm (**b**).

References

1. Selivanova D. G.;Maiorova O.A.; Shklyaeva E.V.; Abashev G.G. *J. Org. Chem*. **2015,** *51*, No 5, 735−739. doi: 10.1134/S1070428015050267
2. Deane, F. M.; Miller, C. M.; Maguire, A. R.; McCarthy, F. O. *J. Heterocyclic Chem.* **2011**, *48*, 814−823. doi: 10.1002/jhet.598
3. Herbivo, C.; Comel, A.; Kirsch, G.; Raposo, M. M. M. *Tetrahedron*. **2009**, *65*, 2079−2086. doi: 10.1016/j.tet.2008.12.078
4. Bushueva, A. Yu.; Shklyaeva, E. V.; Abashev, G. G. *Mendeleev Commun.* **2009**, *19*, 329−331. doi: 10.1016/j.mencom.2009.11.012
5. Varga, L.; Nagy, T.; Kovesdi, I.; Benet-Buchholz, J.; Dorman, G.; Urge, L.; Darvas, F. *Tetrahedron*. **2003**, *59*, 655−662. doi: 10.1016/S0040-4020(02)01560-0
6. Nakazaki, J.; Chung, I.; Matsushita, M. M.; Sugawara, T.; Watanabe, R.; Izuoka, A.; Kawada, Y. *J. Mater. Chem.* **2003**, *13*, 1011–1022. doi: 10.1039/b211986b
7. Kamlet, M. J.; Abboud, J.-L. M.; Abraham, M. H.; Taft, R. W. *J. Org. Chem.* **1983**, *48*, 2877−2887. doi: 10.1021/jo00165a018

3-{9-[4-(4-Methoxyphenoxy)butyl]-9*H*-carbazol-3-yl}-1-(5-{9-[4-(4-methoxyphenoxy)butyl]-9*H*-carbazol-3-yl}thiophen-2-yl)prop-2-en-1-one (6a)

^{1}H NMR (300 MHz, $CDCl_3$)

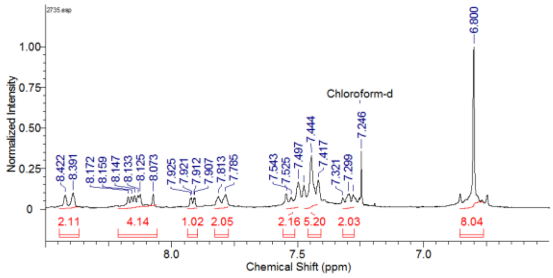

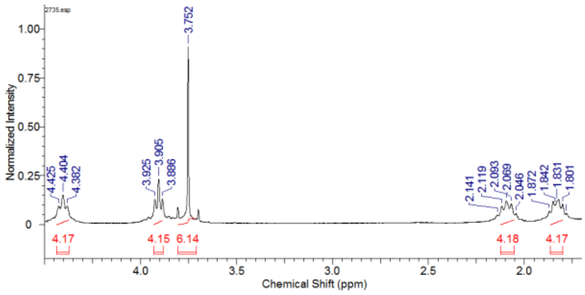

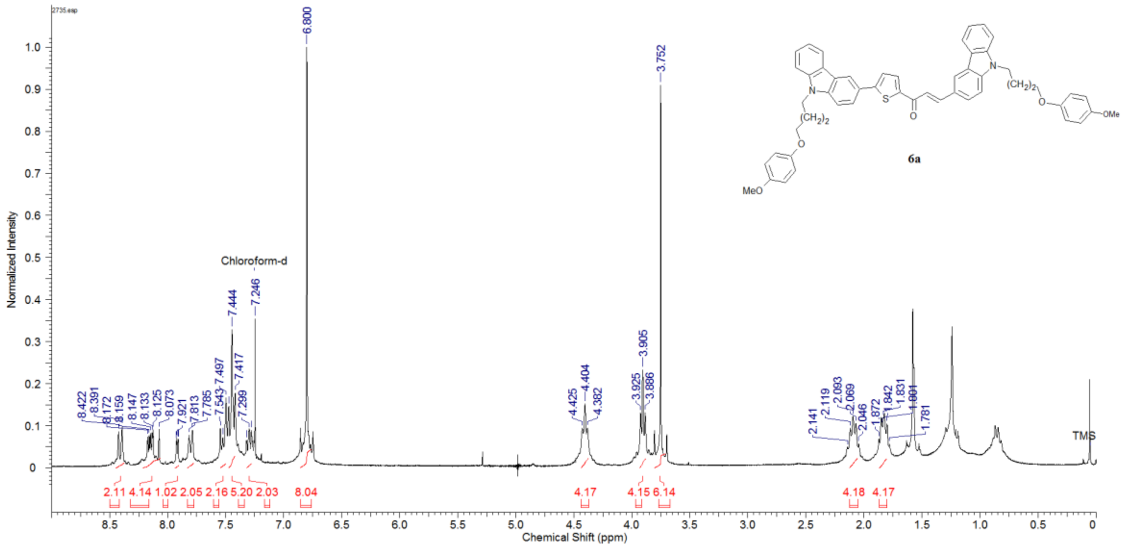

^{13}C (400 MHz)

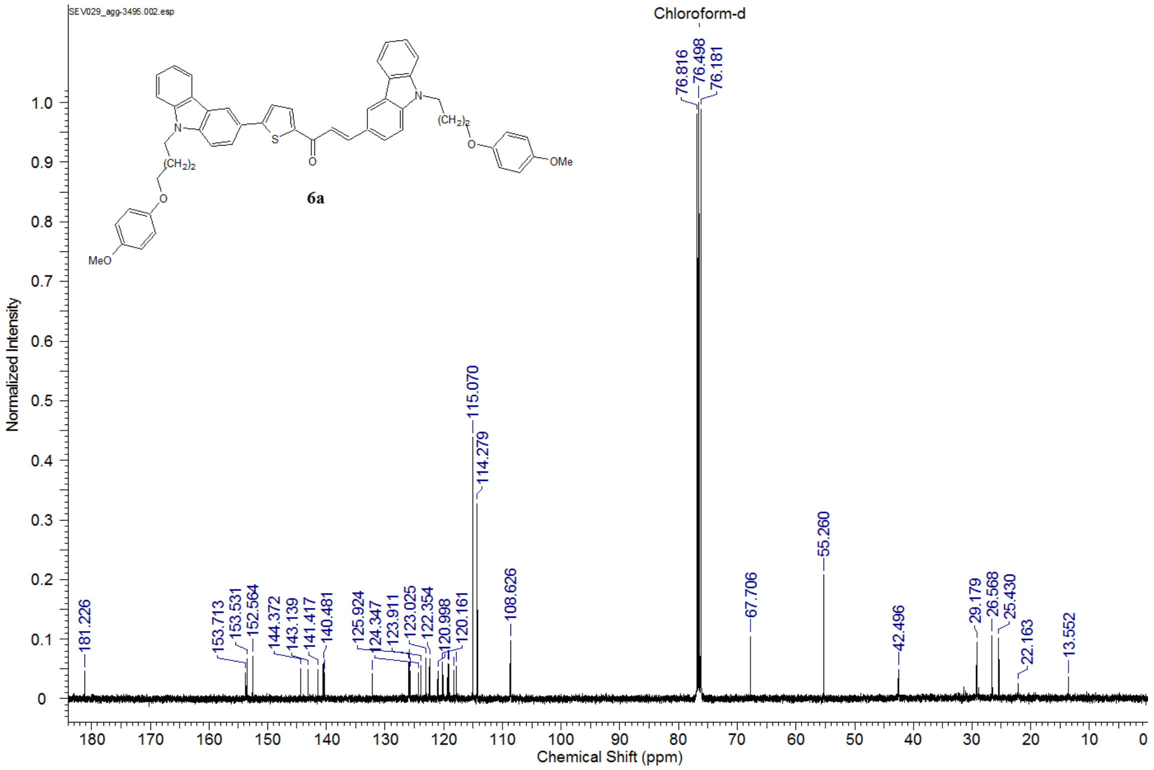

3-{9-[6-(4-Methoxyphenoxy)hexyl]-9*H*-carbazol-3-yl}-1-(5-{9-[6-(4-methoxyphenoxy)hexyl]-9*H*-carbazol-3-yl}thiophen-2-yl)prop-2-en-1-one (6b)

^{1}H NMR (300 MHz, $CDCl_3$)

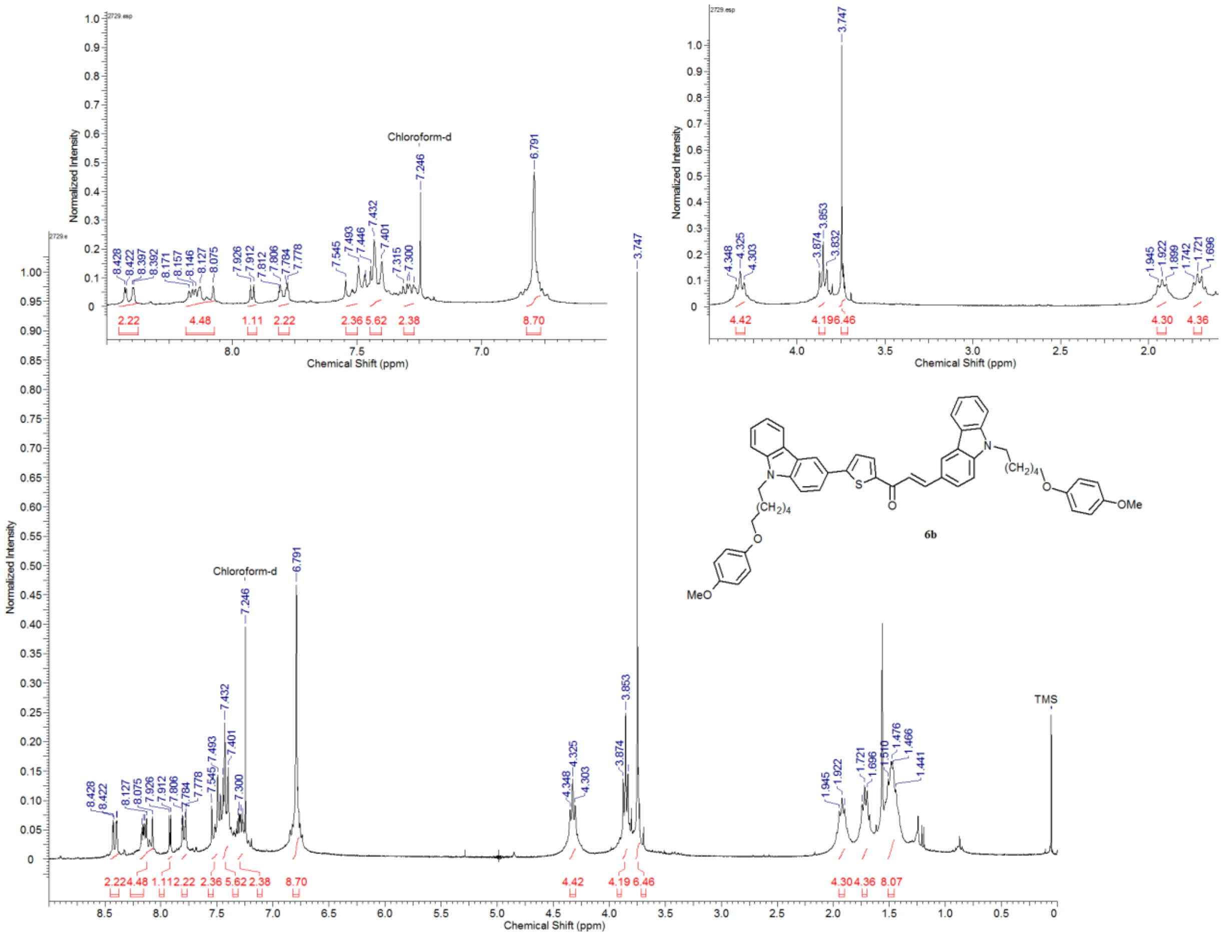

^{13}C (400 MHz)

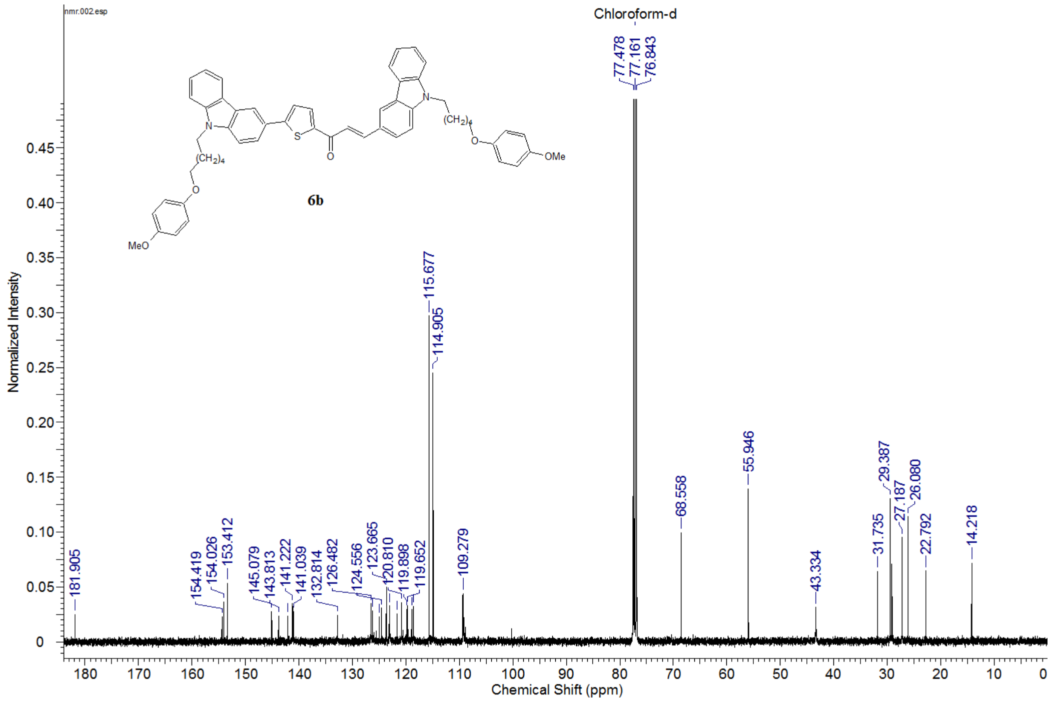

4-{9-[4-(4-Methoxyphenoxy)butyl]-9*H*-carbazol-3-yl}-6-(5-{9-[4-(4-methoxyphenoxy)butyl]-9*H*-carbazol-3-yl}thiophen-2-yl)pyrimidine-2-amine (7a)

^{1}H NMR (300 MHz, $CDCl_3$)

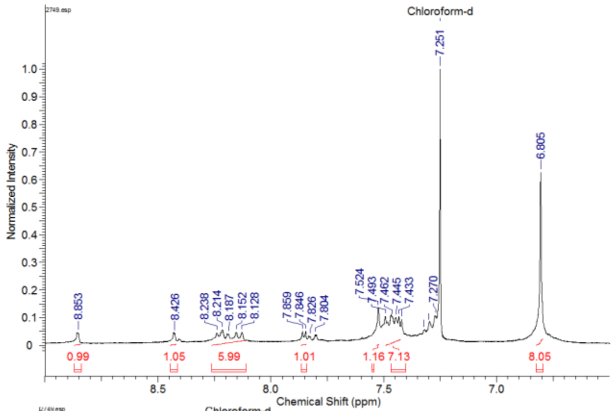

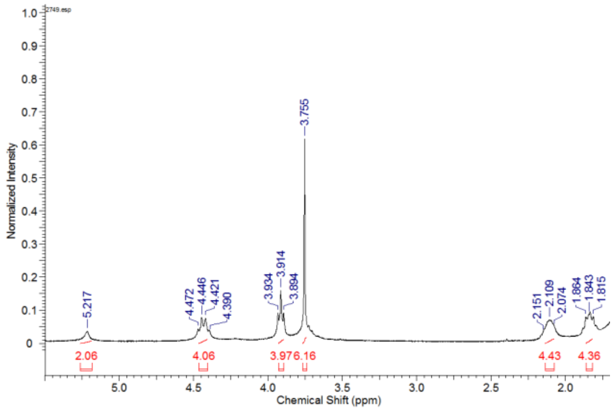

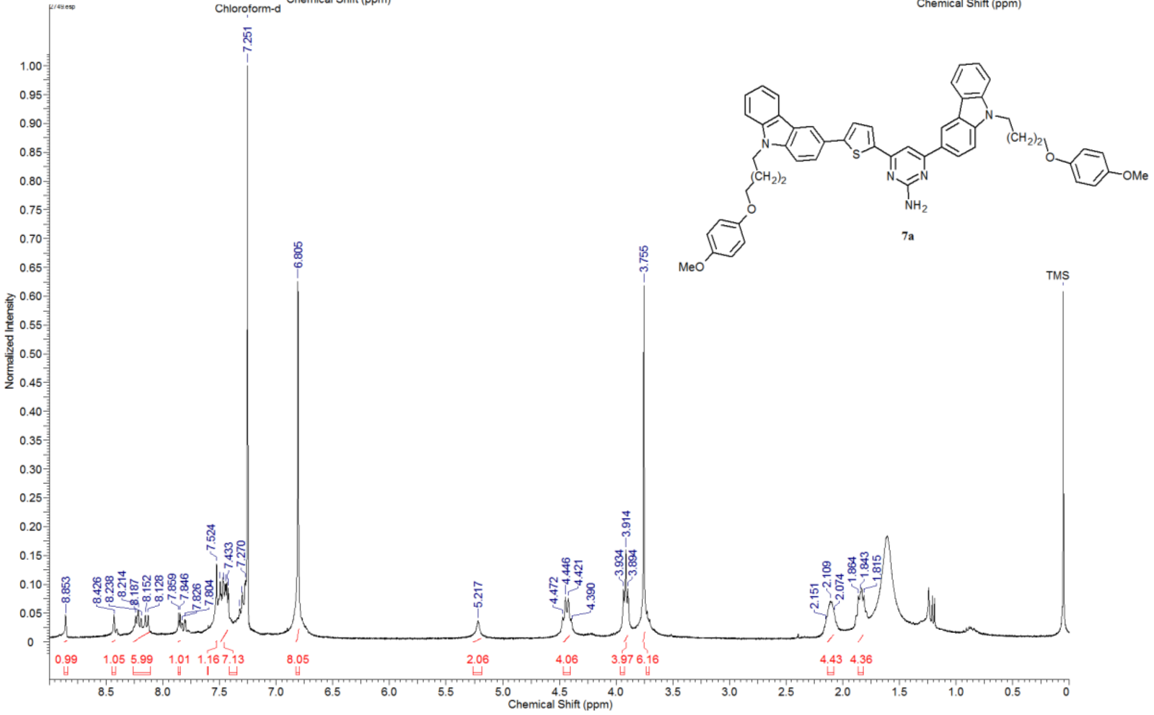

4-{9-[6-(4-Methoxyphenoxy)hexyl]-9*H*-carbazol-3-yl}-6-(5-{9-[6-(4-methoxyphenoxy)hexyl]-9*H*-carbazol-3-yl}thiophen-2-yl)pyrimidine2-amine (7b)

^{1}H NMR (300 MHz, $CDCl_3$)

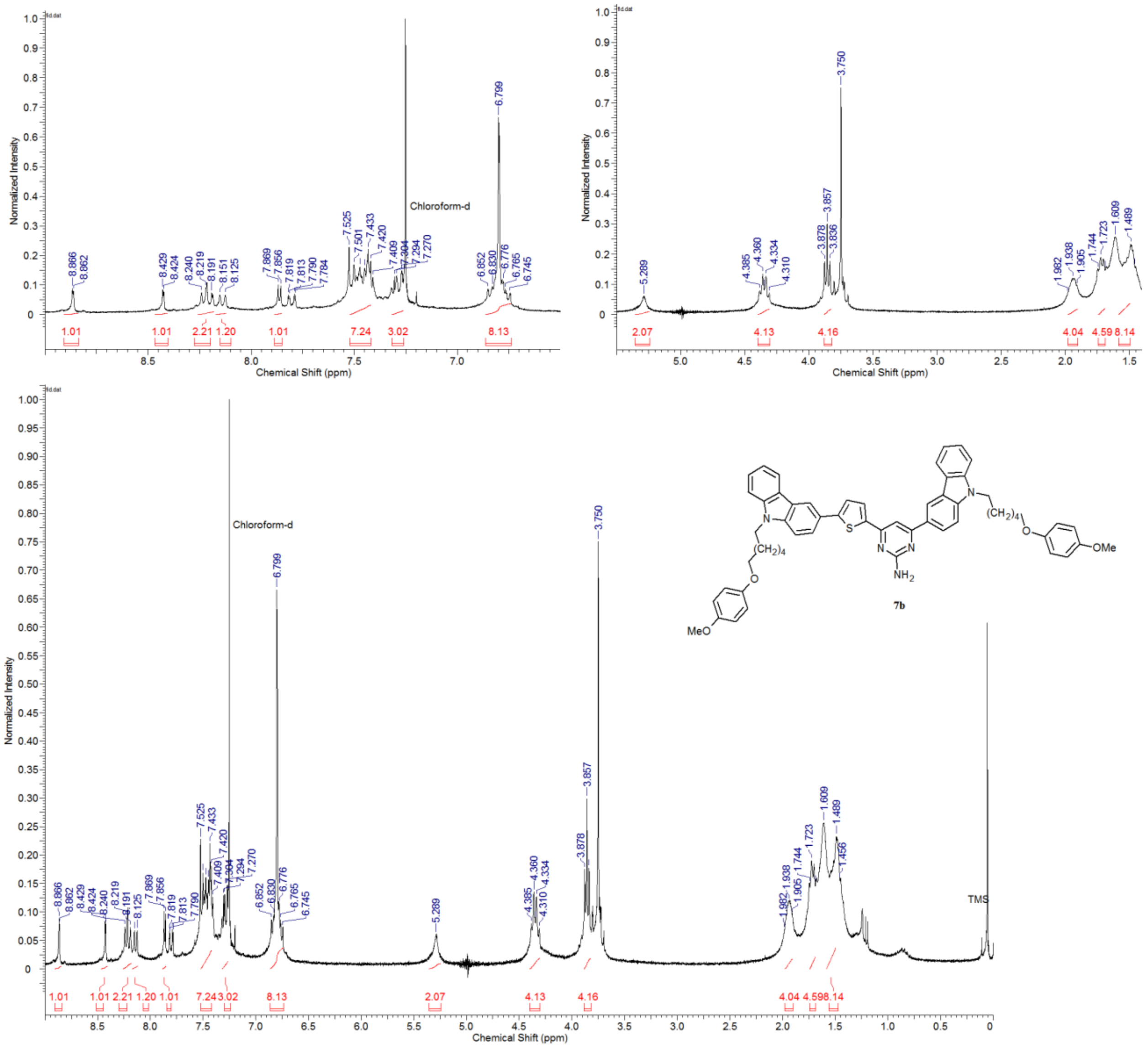

4-{9-[4-(4-Methoxyphenoxy)butyl]-9*H*-carbazol-3-yl}-2-(1H-pyrrol-1-yl)-6-(5-{9-[4-(4-methoxyphenoxy)butyl]-9*H*-carbazol-3-yl}thiophen-2-yl)pyrimidine (8a)

^{1}H NMR (400 MHz, CDCl$_3$)

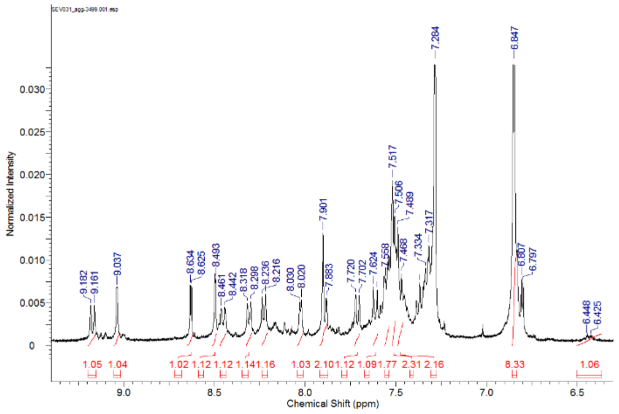

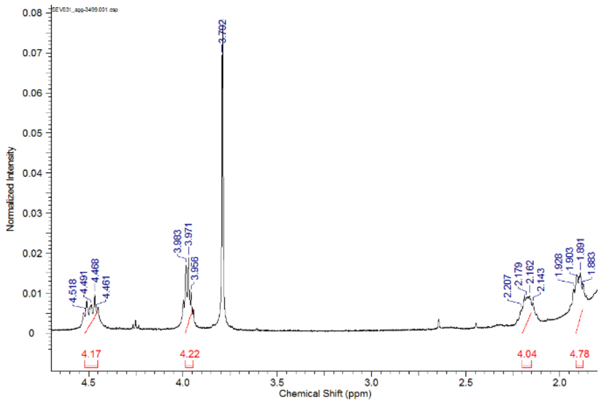

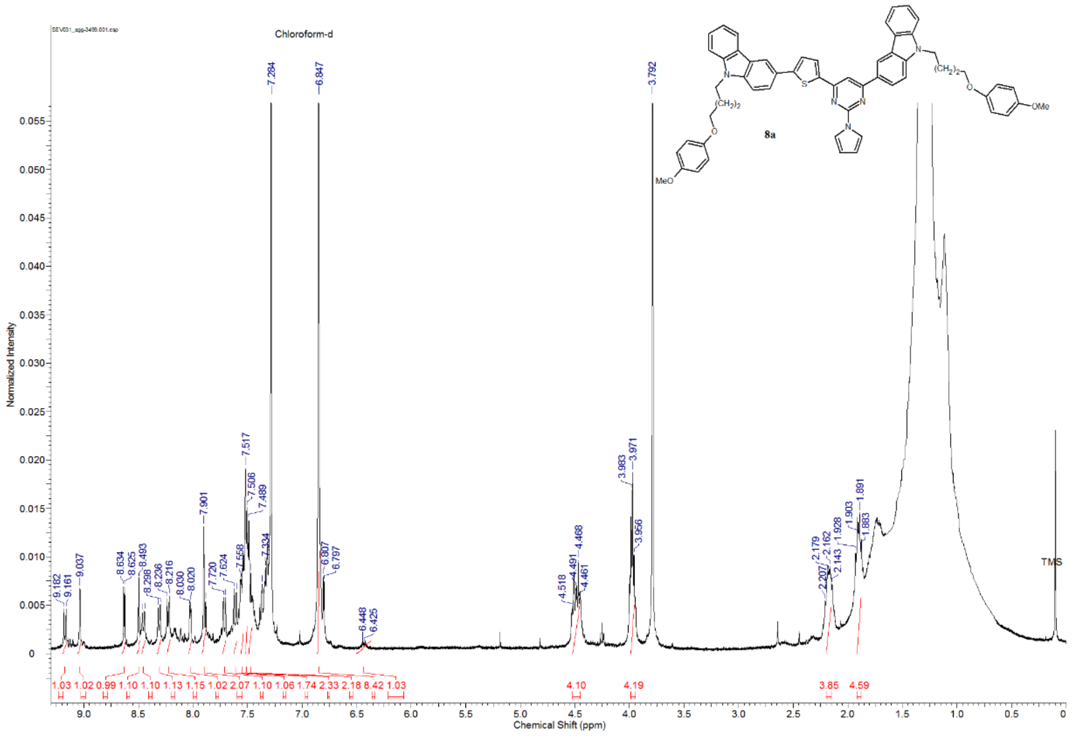

4-{9-[6-(4-Methoxyphenoxy)hexyl]-9*H*-carbazol-3-yl}-2-(1H-pyrrol-1-yl)-6-(5-{9-[6-(4-methoxyphenoxy)hexyl]-9*H*-carbazol-3-yl}thiophen-2-yl)pyrimidine (8b)

^{1}H NMR (300 MHz, $CDCl_3$)

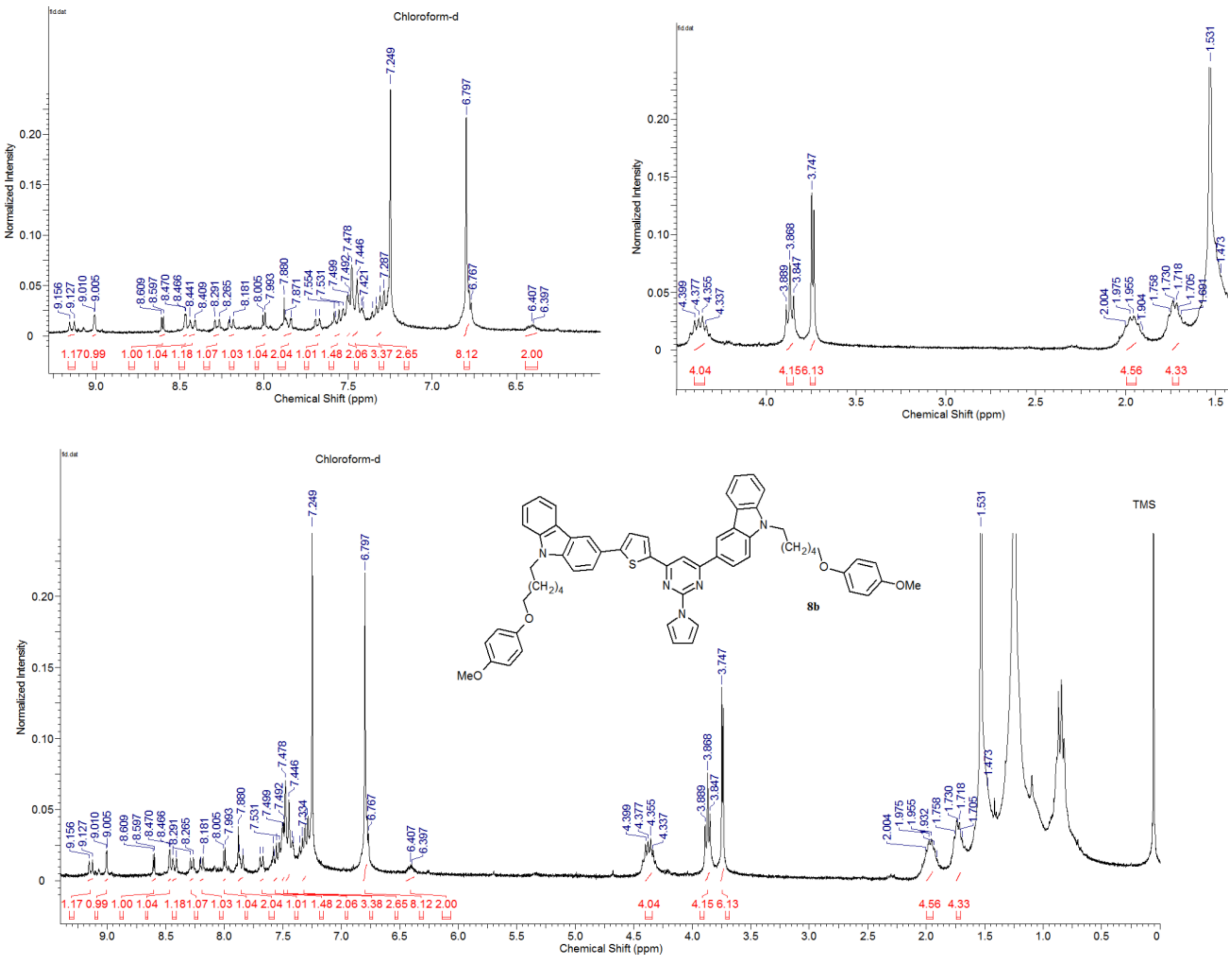

Supplement: File 1 — Synthetic procedures and characterization data for all compounds 1–8 (a,b). [file Beilstein_J_Org_Chem-13-1583-s001.pdf]
